# A Review: Absolute Linear Encoder Measurement Technology

**DOI:** 10.3390/s25195997

**Published:** 2025-09-29

**Authors:** Maqiang Zhao, Yuyu Yuan, Linbin Luo, Xinghui Li

**Affiliations:** Tsinghua Shenzhen International Graduate School, Tsinghua University, Shenzhen 518055, China; zhaomq24@mails.tsinghua.edu.cn (M.Z.); yuanyy24@mails.tsinghua.edu.cn (Y.Y.); luolb24@mails.tsinghua.edu.cn (L.L.)

**Keywords:** absolute linear encoder, displacement measurement, coding technology, error analysis

## Abstract

Absolute linear encoders have emerged as a core technical enabler in the fields of high-end manufacturing and precision displacement measurement, owing to their inherent advantages such as the elimination of the need for homing operations and the retention of position data even upon power failure. However, there remains a notable scarcity of comprehensive review materials that can provide systematic guidance for practitioners engaged in the field of absolute linear encoder measurement technology. The present study aims to address this gap by offering a practical reference to professionals in this domain. In this research, we first systematically delineate the three fundamental categories of measurement principles underlying absolute linear encoders. Subsequently, we analyze the evolutionary trajectory of coding technologies, encompassing the design logics and application characteristics of quasi-absolute coding (including non-embedded and embedded variants) as well as absolute coding (covering multi-track and single-track configurations). Furthermore, we summarize the primary error sources that influence measurement accuracy and explore the operational mechanisms of various types of errors. This study clarifies the key technical pathways and existing challenges associated with absolute linear encoders, thereby providing practitioners in relevant fields with a decision-making guide for technology selection and insights into future development directions. Moving forward, efforts should focus on achieving breakthroughs in critical technologies such as high fault-tolerant coding design, integrated manufacturing, and error compensation, so as to advance the development of absolute linear encoders toward higher precision, miniaturization, cost reduction, and enhanced reliability.

## 1. Introduction

In modern industrial manufacturing, precision measurement, semiconductor processing, and advanced equipment manufacturing, accurate workpiece positioning and high-precision displacement measurement technologies are key enablers for automation control and intelligent manufacturing [1,2,3,4,5,6,7]. In the relevant technical system, grating encoders have emerged as core sensing components, thanks to their outstanding signal stability [8,9,10], wide dynamic bandwidth [11,12,13], and excellent integration compatibility [14,15,16]. With the continuous expansion of requirements for degrees of freedom (DOF) in precision measurement scenarios [17,18,19,20], the technical form of grating encoders has evolved from the early linear encoders [21] to multi-DOF ones, gradually forming a product system covering 2D planar measurement [22,23,24], 3D spatial positioning [25,26,27,28], and even 6-DOF pose detection [29,30,31,32,33].

Among the diverse technical branches of grating encoders, linear encoders, as the core form in their early development stage, were not only the foundation for the subsequent technological iteration of multi-DOF encoders but also have long occupied an irreplaceable position in modern industrial measurement systems due to their advantages of simple structures, high measurement accuracy, stable signal output, fast response speeds, and strong anti-interference capabilities [22,34,35,36,37,38,39,40,41,42,43,44,45,46]. Absolute linear encoders, in particular, are gradually replacing traditional incremental linear encoders as the mainstream solution for high-end motion control systems because they provide real-time absolute position information without requiring homing and do not lose position data after power-off [47].

**Figure 1:**
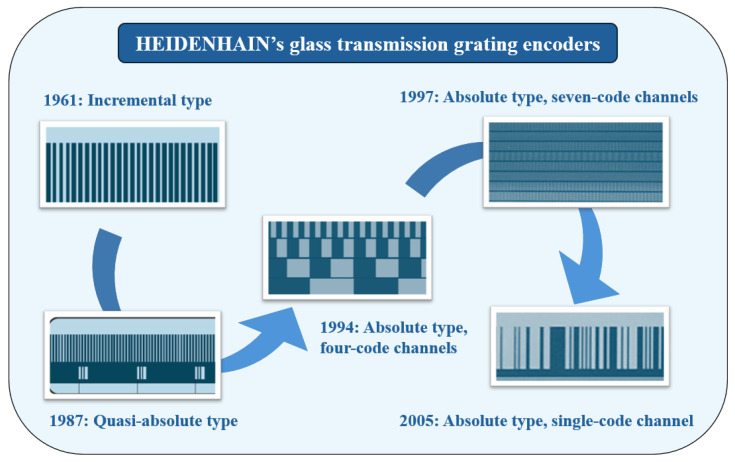
The development history of HEIDENHAIN’s glass transmission linear encoders.

Functionally, linear encoders have evolved from incremental to absolute measurement and from manual digital display applications to CNC system integration. Figure 1 presents the evolution of HEIDENHAIN’s glass transmission linear encoders. HEIDENHAIN began studying multi-track absolute measurement scales in 1994, although the early multi-track encoding products were limited in market adoption due to challenges in grating replication, assembly, and reliability.

As can be seen, in the technological evolution of absolute linear encoders, the differentiation between quasi-absolute encoding and absolute encoding constitutes a key developmental thread. In the early stage, limited by process complexity, the industry adopted the quasi-absolute linear encoder scheme with “incremental track + aperiodic auxiliary track” to achieve absolute positioning, which requires homing operation [48,49]. With breakthroughs in grating fabrication and decoding algorithms, absolute linear encoders now realize real-time absolute position output without the need for homing through multi-track parallel encoding (such as natural binary code [50], Gray code [51], and vernier code [52,53]) or single-track pseudorandom sequences [54,55,56,57], thus addressing the limitations of quasi-absolute linear encoders that lose reference after power failure.

In developed countries, research on high-precision optical linear encoders, especially absolute types, began relatively early, with mature technical routes and well-established industrial systems. Representative companies include HEIDENHAIN (Germany), Renishaw (UK), FAGOR (Spain), and MITUTOYO (Japan), all of which have developed series products. In China, research on high-precision optical linear encoders began slightly later but has progressed rapidly. Since the 1960s–1970s, institutions such as the Changchun Institute of Optics and Fine Mechanics, the Chengdu Institute of Optoelectronics, and Zhejiang University have been conducting related studies. In recent years, research units including the Changchun Institute of Optics, Guangzhou Xinhao, Langfang Leige, and Zhuhai Yixin have made breakthroughs in accuracy improvement, packaging, and absolute coding schemes. Notably, the Changchun Institute of Optics developed China’s first industrializable single-track absolute linear encoder in 2009, marking a significant milestone. Despite China being a major producer of displacement sensors, high-end sensor core technologies are still mainly imported. Domestic high-performance grating encoders still lag behind international advanced levels in terms of accuracy, reliability, and industrialization [58,59,60].

Absolute linear encoders are essential for precise positioning and high-precision displacement measurement in modern manufacturing. Recent research has focused on high-accuracy 3D reconstruction and phase measurement networks [61,62]. Reviews on precision positioning and measurement technologies provide a comprehensive understanding of the global research status [3,43]. Currently, in the field of absolute encoder measurement technology, there is still a relative scarcity of summary materials that can serve as references for practitioners and provide systematic guidance. This paper aims to provide a reference for the development of absolute linear encoder measurement technology and presents a systematic study focusing on the measurement principles, encoding methods, and performance evaluation approaches of absolute linear encoders. The structure is as follows:

(1) Introduction, including the background and significance of the research, a review of domestic and foreign research, and an outline of the research content and thesis structure.
(2) Fundamental theories of absolute optical linear encoders, including measurement principles based on Moiré fringes, diffraction gratings, and image processing; coding and decoding technologies, including traditional absolute coding, serial communication protocols, and absolute decoding; and performance indicators, such as the measuring range, accuracy, pitch and resolution, motion speed, and stability. Meanwhile, a selection guideline for absolute linear encoders is presented.
(3) Study of quasi-absolute coding methods, detailing non-embedded and embedded coding principles, structural characteristics, and application scenarios.
(4) Systematic discussion of absolute coding techniques, including multi-track absolute coding (natural binary, Gray code, matrix code, vernier code, and combinations of absolute and incremental tracks) and single-track absolute coding (hybrid coding, displacement continuous coding, and pseudorandom sequence coding).
(5) Error analysis in absolute linear encoder measurement, including manufacturing process-related errors, optical system errors, signal processing and coding errors, and environmental interference, with discussions of causes and impacts.
(6) Conclusions, summarizing the research, discussing current technological trends, and exploring potential future developments and breakthroughs for absolute optical linear encoders.

## 2. Fundamental Theory of Absolute Linear Encoder Measurement

### 2.1. Basic Principles of Linear Encoder Measurement

A linear encoder is a high-precision linear displacement sensor that uses a high-accuracy long grating as the measurement reference. It converts optical signals into electrical signals through a photoelectric sensor and processes these electrical signals to ultimately obtain position information. Compared with other linear displacement sensors (such as magnetic scales, ball scales, and laser interferometers), linear encoders offer superior overall performance in terms of measurement accuracy, resolution, reliability, environmental adaptability, and cost. Therefore, linear encoders are widely used in digital displays, CNC machine tools, and measurement instruments [63,64,65,66,67]. In recent years, related research has included new fabrication methods for imaging devices (such as two-photon polymerization lithography for imaging optics), as well as three-dimensional reconstruction and soft sensor modeling based on machine learning models. These efforts have collectively advanced the measurement system in terms of resolution, dynamic range, and robustness and provided a methodological basis for studying the application of absolute encoders in complex industrial scenarios [68,69,70,71].

Linear encoders are mainly divided into two types: incremental and absolute. Incremental encoders generate pulse signals by detecting changes in Moiré fringes, calculate the relative displacement based on pulse accumulation, require zero return upon startup, feature a simple structure and low cost, and are suitable for conventional displacement measurement scenarios. Absolute linear encoders, on the other hand, directly output absolute position data through unique encoded patterns on the scale, eliminating the need for zero return and retaining position information even when power is off. They offer higher precision and reliability, making them suitable for precision applications with high requirements for the initial position accuracy [72].

**Figure 2:**
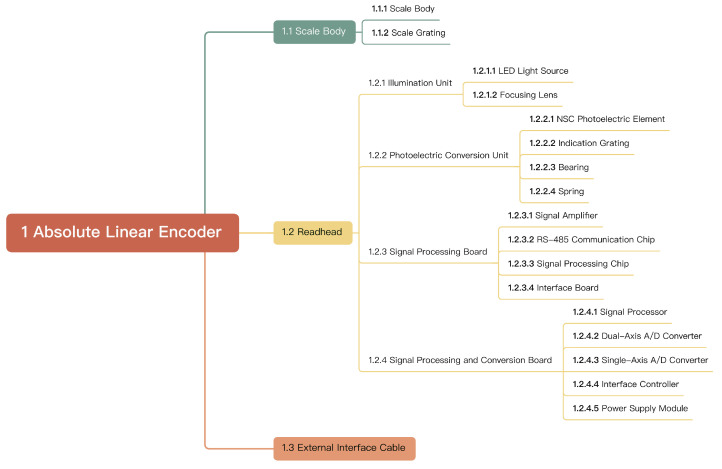
Functional block diagram of an absolute linear encoder.

Based on the mechanism of optical signal generation, linear encoders can be further categorized into absolute measurement technology based on Moiré patterns, absolute measurement technology based on diffraction gratings, and absolute measurement technology based on image processing [42,73,74]. The functional block diagram of an absolute linear encoder is shown in Figure 2. In the fields of interference and optical sensing, there have been improved data processing algorithms, composite beam (multiple beam) interference theories, and second harmonic/third harmonic and nonlinear optical angle measurement methods, as well as research on enhancing signal calculation with artificial intelligence. These achievements provide specific implementation paths for achieving a high resolution, large range, and high dynamic response [11,75,76,77,78,79,80,81,82,83,84,85,86].

Coding and decoding techniques involve traditional absolute coding, serial communication protocols, and absolute decoding. Signal processing approaches such as linearized converters and ratiometric interpolators have been proposed to enhance the absolute measurement accuracy [87,88]. Key performance indicators include the measurement range, resolution, speed, and stability [89,90].

#### 2.1.1. Principles of Linear Encoder Measurement Based on Moiré Fringes

**Figure 3:**
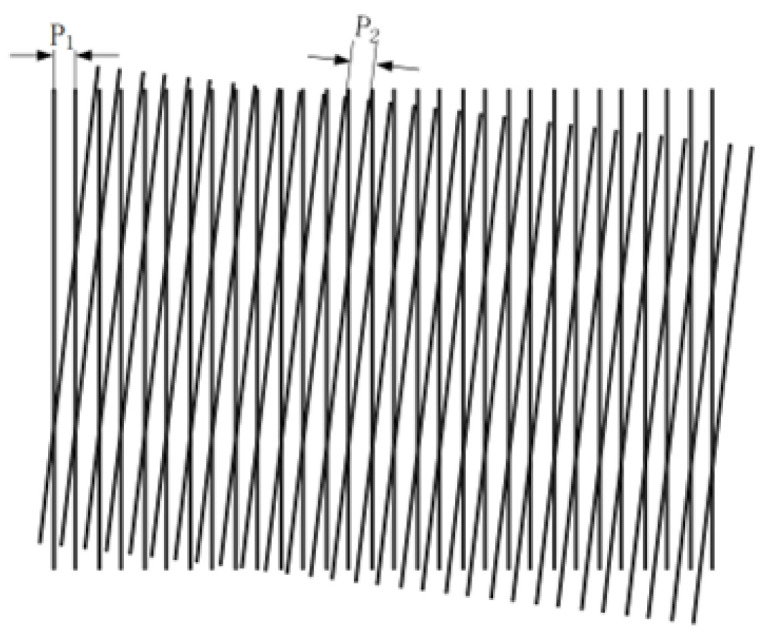
Superposition of two gratings.

In recent years, the Moiré fringe method for absolute position alignment measurement has attracted increased attention [91,92]. A Moiré-based linear encoder consists of a light source, lenses, a grating pair (scale grating and index grating), and photosensitive elements. Its working principle is to superimpose a scale grating and an index grating with exactly the same grating constant, with a slight angle between their grating lines. When light is incident, bright bands appear where the lines of the two gratings coincide due to interference, and dark bands appear where the lines are offset. These alternating bright and dark bands form the Moiré fringes, as shown in Figure 3.

Methods for analyzing Moiré fringes include geometric optics, diffraction optics, and Fourier analysis. In CNC systems, the incremental period of linear encoders is generally 20 μm, much larger than the LED emission wavelength (typically around 850 nm for infrared LEDs), so the shadow principle of geometric optics can be used to explain the phenomenon.

As shown in Figure 3, the two gratings have grating pitches $P_{1}$ and $P_{2}$, and the angle between them is θ. The diagonal of the parallelogram formed by the transparent areas of the two gratings corresponds to twice the Moiré fringe width, 2W. Using planar geometry, it can be derived that

(1) When $P_{1}\neq P_{2}$ and θ≠0, the fringes are called oblique Moiré fringes;
(2) When $\theta <10^{-3}$, the Moiré fringes are approximately perpendicular to the grating lines, called transverse Moiré fringes;
(3) When $P_{1}\neq P_{2}$ and θ=0, the fringes move parallel to the grating lines, called longitudinal Moiré fringes;
(4) When $P_{1}=P_{2}$ and θ=0, the fringe width tends to infinity, called strobe Moiré fringes.

In practical linear encoder applications, strobe Moiré fringes are usually used to generate periodic signals, with the relative displacement between the two gratings as the variable. Photocells detect the Moiré fringes formed by scale and index gratings. Four identical photocells are used, each with an index grating fixed on top. The relative positions of the four index gratings are

(1) $$\begin{array}{cc} W_{1} & =(N_{1}+0.25)\times P \end{array}$$

(2) $$\begin{array}{cc} W_{2} & =(N_{2}+0.5)\times P \end{array}$$

(3) $$\begin{array}{cc} W_{3} & =(N_{3}+0.75)\times P \end{array}$$

where *P* is the grating pitch and $N_{1},N_{2},N_{3}$ are positive integers.

**Figure 4:**
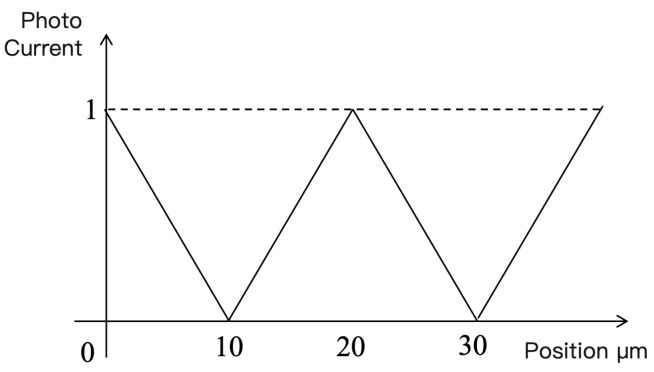
Relationship between photocurrent and relative displacement of grating pair.

Assuming parallel light illumination and zero spacing between the grating pair, the photocurrent generated by each photocell is proportional to the overlapping area of the transparent parts of the two gratings. Therefore, the photocurrent is proportional to the relative displacement of the grating pair, forming a linear function, as shown in Figure 4.

**Figure 5:**
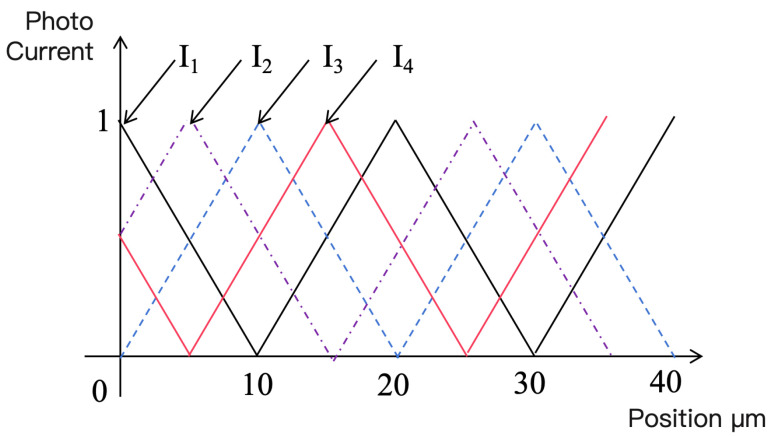
Photocurrent variation caused by relative movement of grating pair. ($I_{1}$–$I_{4}$ are four signals with a phase difference of 90° between each other. The black line represents $I_{1}$, the purple line represents $I_{2}$, the blue line represents $I_{3}$, and the red line represents $I_{4}$).

In practice, optical filtering can be used to convert the triangular waveform in Figure 5 into a sine wave, producing four sine signals with phase differences of 90°. To remove common-mode signals, signals with a 180° phase difference are subtracted, resulting in two orthogonal sine signals. By counting and interpolating these two orthogonal signals, the relative displacement between the index and scale gratings can be obtained.

Strobe Moiré fringes are very convenient for use in linear encoders. From Equations (1)–(3), it can be seen that $N_{1},N_{2},$ and $N_{3}$ only need to be integers to obtain four-phase photocurrent signals with successive 90° phase differences. Therefore, the arrangement of the four windows on the index grating is relatively flexible and easy to implement. Consequently, most early foreign and current domestic linear encoder products adopt strobe Moiré fringes.

**Figure 6:**
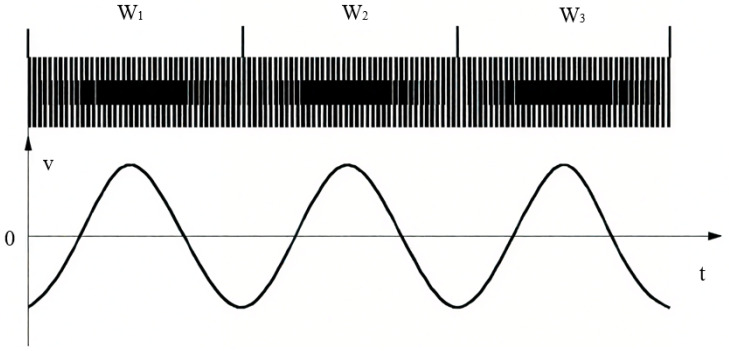
Schematic diagram of longitudinal Moiré fringes in linear encoders.

For longitudinal Moiré fringes, as shown in Figure 6, there are specific requirements for the size and arrangement of the photocells. Let the grating pitch of the index grating be $P_{1}$ and that of the scale grating be $P_{2}$, and let the period of the Moiré fringe be *D*. Since $P_{1}$ and $P_{2}$ are very close, the relationship among them is

(4) $$D=\frac{mP_{1}P_{2}}{|P_{1}-P_{2}|}$$

where *m* is the difference in the number of grating lines within the Moiré fringe range. When the index grating moves by one grating pitch relative to the scale grating, the Moiré fringe moves by one period. The displacement of the Moiré fringe is proportional to the movement of the index grating. The magnification factor of the Moiré fringe is

(5) $$N=\frac{D}{P_{2}}=\frac{mP_{1}}{|P_{1}-P_{2}|}$$

From the above equation, it can be seen that, when $P_{1}-P_{2}$ is very small, the magnification *N* can be very large. Therefore, detecting the Moiré fringes, which are magnified many times, is easier than detecting the index grating itself, improving the resolution of the measurement. The larger the width of the fringe *D*, the closer the two grating pitches are and the more difficult it is to ensure the precision of manufacturing. For longitudinal Moiré fringes, the movement direction of the fringes is the same as that of the two gratings forming the fringes [93]. In recent years, companies like HEIDENHAIN have successfully applied longitudinal Moiré fringes by designing large-area gratings with excellent results. Photodiode arrays can typically receive multiple sets of longitudinal Moiré fringes; every four adjacent photocells capture one full Moiré fringe cycle. Receiving multiple sets allows error averaging and mitigates local defects.

**Figure 7:**
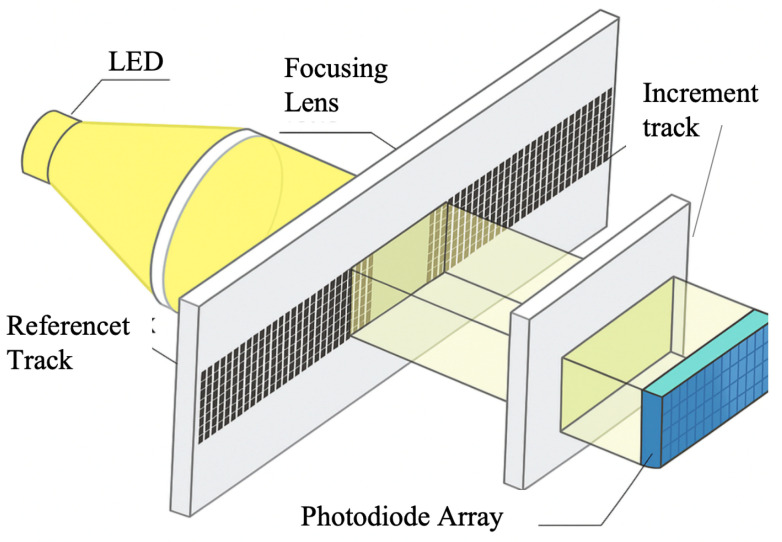
Application method of longitudinal moiré fringes in linear encoders.

With the rapid development of microelectronics, some international encoder manufacturers (e.g., HEIDENHAIN) have successfully applied longitudinal Moiré fringes in linear encoder design by customizing photodiode arrays with specific spacing and quantities. As shown in Figure 7, this type of linear encoder uses LEDs as light sources and adjusts three key parameters—the pitch of the scale grating, the pitch of the index grating, and the light spot size—to match a custom photodiode array for efficient signal acquisition.

Linear encoders convert the light intensity signals of Moiré fringes varying with displacement into electrical signals via photosensitive elements, thereby indirectly measuring the displacement. This method has the following advantages [94]:

(1) The magnification effect of Moiré fringes allows high-sensitivity displacement measurement;
(2) Moiré fringes average out grating line errors, effectively suppressing local line errors and achieving higher accuracy compared to direct measurement methods;
(3) Non-contact measurement is possible, improving system stability and service life.

High-quality Moiré fringes are a key prerequisite for achieving high-resolution measurement. However, higher-order harmonics in the Moiré fringes can significantly affect the sinusoidality and orthogonality of incremental signals. Therefore, multi-window spatial filtering techniques are widely used to suppress higher-order harmonic components, improving the signal quality and ensuring higher measurement accuracy and a greater resolution in the linear encoder system [95].

In the aforementioned Moiré-based measurement methods, relatively large-period optical gratings (typically on the order of 20 μm) are commonly employed. Such gratings generate approximately linear optical responses upon transmission or reflection, which facilitates the formation of clear Moiré fringes. However, large-period gratings suffer from inherent resolution limitations, with the optical sensitivity typically constrained to the micrometer scale.

When the grating period is reduced to the submicrometer range (typically 0.1–1 μm), a pronounced Talbot self-imaging effect emerges in the diffraction near-field. In other words, the Talbot effect can be regarded as a special case of the Moiré method in the small-period limit. Under conditions of spatially coherent illumination, the periodic structure is periodically reconstructed along the propagation direction, with the characteristic length determined by the Talbot length:

(6) $$z_{T}=\frac{2d^{2}}{\lambda }$$

where *d* is the grating period and λ is the wavelength of the light source. As the grating period decreases, the self-imaging fringes become more densely distributed in space, resulting in significantly enhanced sensitivity to minute displacements and thus overcoming the resolution bottleneck of conventional large-period Moiré fringes [96,97].

Grating measurement based on the Talbot effect exhibits several notable features. First, the self-imaging pattern is highly sensitive to the relative displacement or angular variations of the grating, and, when combined with phase demodulation and sub-pixel interpolation techniques, a nanometer-level resolution can be achieved. For example, a Talbot displacement sensor employing angular-modulated double-layer gratings demonstrated a resolution of 11.23 nm within a 1 mm measurement range [98]. Second, unlike the approximately linear Moiré fringes generated by large-period gratings, small-period gratings produce Talbot fields with periodic diffraction distributions containing higher spatial frequency components. Consequently, photodetector arrays or imaging systems combined with digital signal processing are typically required for accurate decoding [96,99]. In addition, the Talbot effect is highly sensitive to the coherence of the light source and the precision of system alignment. Narrowband lasers or optimized phase grating structures are often used to enhance the self-imaging contrast, with phase gratings offering superior optical efficiency and tolerance to spacing errors compared to amplitude gratings [99].

More specifically, when a spatially coherent beam illuminates a periodic grating, self-images of the grating are formed at fixed intervals along the propagation direction, without the need for an external imaging system. These image planes are known as Talbot planes, with adjacent planes separated by one Talbot length [100]. Notably, at half the Talbot length, the reconstructed image undergoes a lateral shift of λ/2.

In recent years, the Talbot effect has found wide application across various precision measurement fields. For instance, in spectroscopy, methods based on the Fourier transform of the Talbot length enable compact spectral acquisition [101,102,103]; in wavefront sensing [104] and aberration measurement [105], Talbot interferometry has demonstrated high sensitivity; and, in displacement, angular, and tilt measurement, the Talbot effect has likewise enabled high-precision detection [98,106,107]. Therefore, the Talbot effect not only provides a feasible pathway to surpass the resolution limits of traditional Moiré techniques but also opens new avenues for micro- and nanoscale precision metrology and advanced optical sensing.

#### 2.1.2. Principles of Linear Encoder Measurement Based on Diffraction Gratings

**Figure 8:**
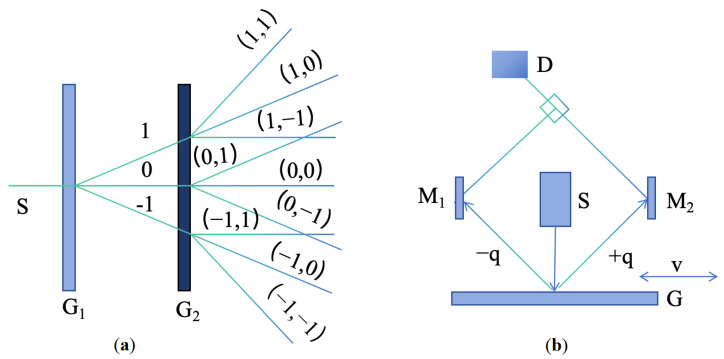
Schematic of diffraction-based grating displacement measurement system. S represents the light source, G the grating, M the mirror, and D the detector. (**a**) Double grating. (**b**) Single grating.

Diffraction gratings with periodic micro- and nano-scale structures have been greatly developed in the field of optical encoders [108,109,110,111,112,113,114,115]. At present, precision nanoscale measurement technologies mainly fall into three categories: time grating sensors, laser interferometers, and grating interferometers [116,117,118,119,120]. Grating interferometers have the outstanding advantage of high sensitivity in displacement measurements [14,23,121,122,123]. A diffraction grating interferometric displacement measurement system measures displacement by using the interference of diffracted light to produce alternating bright and dark fringes [124]. The schematic is shown in Figure 8a,b. When the grating pitch is smaller than 10 μm, it is close to the light source wavelength, and diffraction effects become increasingly significant.

The system in Figure 8a uses a double-grating structure. Light from the source is first diffracted by grating $G_{1}$ and then illuminates the second grating $G_{2}$. $G_{2}$ diffracts the incident light again, and diffracted beams of the same order interfere, forming interference fringes [125]. When the gratings move relative to each other, the brightness of the interference fringes changes, enabling displacement measurement.

Figure 8b shows a single-grating system. Laser light passing through a single grating produces diffraction; the ±*q*-order symmetric diffracted beams interfere, and the interference fringes are detected. According to the Doppler effect [126,127], when the grating moves a distance *s* at velocity *v*, the ±*q* diffracted beams produce an angular frequency difference Δω=4πqv/d. After heterodyne interference, the light intensity *I* relates to displacement *s* as I∼cos(4πqs/d), converting the displacement into an electrical signal. When the ±1 order is used, the system achieves optical frequency doubling.

In grating fabrication, key parameters such as the grating pitch, sub-grating fill factor, etching depth, and buried oxide (BOX) layer thickness should be taken into consideration [128]. In such systems, the grating pitch *d* is a key factor determining the system resolution. Compared to traditional interferometry, the diffracted light itself participates in the interference, significantly enhancing the signal-to-noise ratio. Diffraction grating interferometric displacement measurement systems provide a high resolution, a flexible structure, and strong adaptability. Current research trends include miniaturization, higher scanning speeds and resolutions, improved accuracy and stability, extended measurement ranges, and multi-dimensional measurement capabilities.

**Figure 9:**
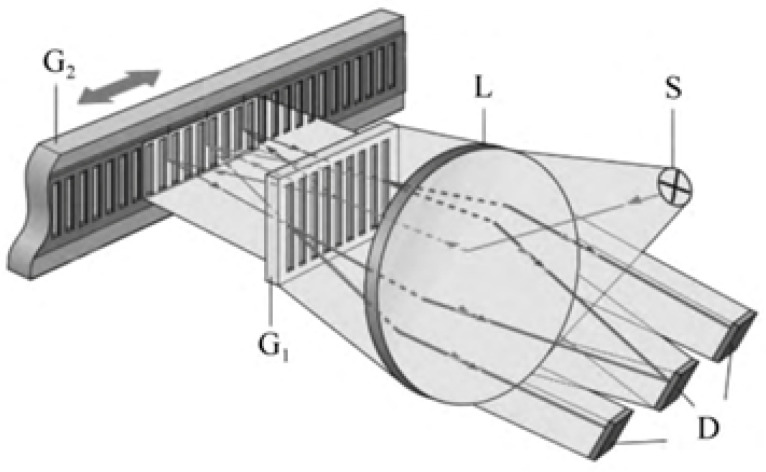
Schematic of diffraction grating interferometer displacement measurement system (HEIDENHAIN).

HEIDENHAIN’s LIP, LIF, and PP series products [129] are diffraction grating interferometric displacement measurement systems, typically using gratings with pitches of 8 μm and 4 μm. Figure 9 shows the system schematic.

**Figure 10:**
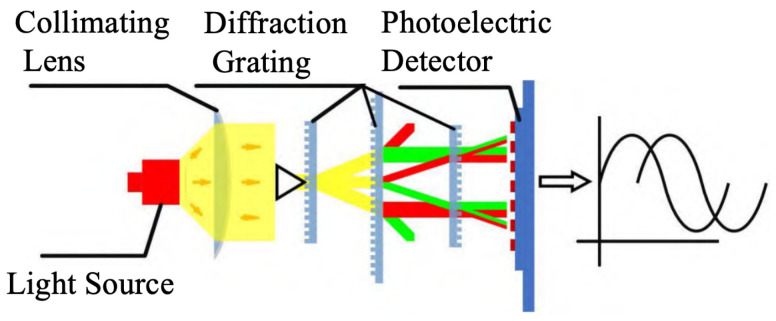
Linear displacement measurement technology based on diffraction gratings [130]. (Red and green lines indicate different diffraction orders forming the sinusoidal signal).

The following shows a transmission-type double-grating interferometric scanning system. Its measurement principle is illustrated in Figure 10. Light from the source is diffracted by the first grating into different orders and then diffracted again by a second grating. The two diffracted beams superimpose to produce interference fringes, which are converted into electrical signals by photoelectric devices. Relative motion between the grating and detector causes phase shifts, enabling the determination of the relative position of the index grating.

**Figure 11:**
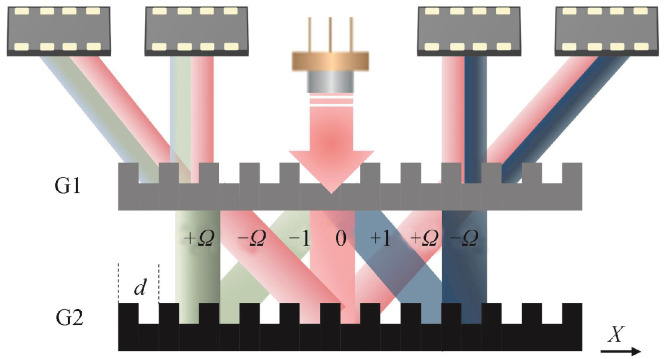
Schematic of double-grating diffraction. (G1: transmission grating; G2: reflection grating; Red, blue, and green lines represent the 0th, +1st, and –1st diffraction orders).

Figure 11 shows a reflective double-grating interferometric scanning system [131]. Its core optical elements are a transmission phase grating $G_{1}$ (reference) and a reflective phase grating $G_{2}$ (measurement). By optimizing the grating pitch, selecting the diffraction order, and improving the diffraction efficiency, three-step phase shifting is possible, enabling the miniaturization of the readhead. The optical path is as follows: collimated light from a semiconductor laser illuminates the reference grating $G_{1}$, generating 0- and ±1-order diffracted beams; the beams reflect and diffract from the measurement grating $G_{2}$; reflected light diffracts again through $G_{1}$; beams in the same direction interfere, producing periodic interference signals related to displacement, detected by a photodetector, and output as four phase-shifted signals.

In this double-grating scanning system, the beam undergoes three diffraction events, as shown in Figure 11. Light passing through the reference grating produces 0-, +1-, and −1-order beams incident on the measurement grating. According to the grating phase shift theorem and Doppler effect, the relative displacement *X* of the measurement grating along the *X*-axis causes the +1-order diffracted light to experience a phase shift of +Ω and that of the −1 order to experience a phase shift of −Ω. This phase shift Ω is positively correlated with the displacement *X*, grating pitch *d*, and diffraction order [132]:

(7) $$\begin{array}{c} \Omega =2\pi \times \frac{X}{d} \end{array}$$

The relationship between the interference intensity of the three beams and the relative displacement can be expressed as

(8) $$\begin{array}{cc} I_{1} & =a+b\cos\left(\frac{4\pi X}{d}+2\varphi \right) \end{array}$$

(9) $$\begin{array}{cc} I_{2} & =a+b\cos\left(\frac{4\pi X}{d}\right) \end{array}$$

(10) $$\begin{array}{cc} I_{3} & =a+b\cos\left(\frac{4\pi X}{d}-2\varphi \right) \end{array}$$

where $I_{1},I_{2},I_{3}$ are the intensities of the three interference beams, *a* is the DC component, *b* is the amplitude of the interference signal, and *X* is the displacement. Solving the three equations simultaneously removes the influence of the DC and AC amplitudes, yielding the displacement *X* [133]. Inside the readhead, photocells receive three cosine signals with 2φ phase differences, convert them to electrical signals, amplify them, and process them in subsequent circuits to obtain the desired displacement signal.

Technologies based on interferometry exhibit extremely high precision in phase measurement (exceeding 1/100 of the wavelength) and enable the direct acquisition of wavefront aberrations under very large apertures; however, the relative complexity of interferogram decoding and the sensitivity of measurement equipment to vibrations have hindered their widespread application [134]. In addition to grating-based methods, chromatic confocal technologies have also been widely adopted for high-precision displacement and surface profile measurement. Li et al. provided a comprehensive review of recent advances in chromatic confocal sensors, highlighting their capabilities for non-contact, high-resolution, and multi-dimensional measurement in industrial and microfabrication applications [78].

#### 2.1.3. Principles of Linear Encoder Measurement Based on Image Processing

**Figure 12:**
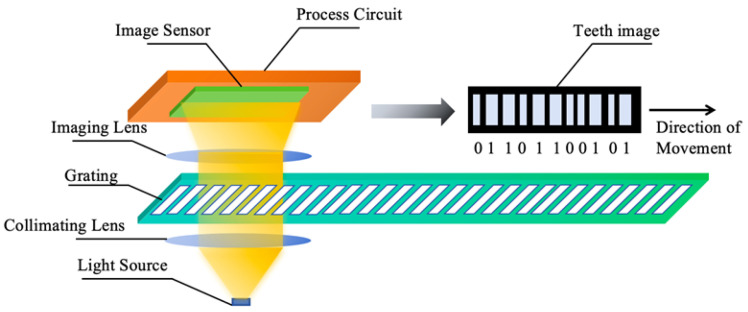
Linear displacement measurement technology based on image processing algorithms.

The measurement principle of linear encoders based on image processing has broad application potential in actual complex scenarios [13]. To overcome the resolution limitations of Moiré fringe measurement and the difficulty in achieving absolute measurement with diffraction gratings, digital image processing algorithms for displacement measurement have received increased attention in recent years. The measurement principle is shown in Figure 12 [130]. The main components include a light source, calibrated grating, imaging lens, and image sensor. During measurement, light emitted from the source passes through the calibrated grating, is magnified by the imaging lens, and reaches the image sensor. The image sensor converts the received optical signal into an electrical signal. Through image processing techniques such as Fourier transforms and filtering, the grating image is obtained. By analyzing the grating image, the current position can be determined. Sub-pixel-level displacement measurement is achieved by interpolating adjacent code elements.

An image sensor consists of a pixel array and readout circuits. Each pixel contains a photodetector that generates a photocharge under illumination, forming a photocurrent. The readout circuit converts this photocurrent into a voltage signal. Popular products feature very high pixel counts, reaching hundreds of millions, with pixel sizes on the micrometer scale. To improve the light transmission efficiency and effectively collect light, some products integrate microlens arrays to focus incoming light.

Currently, there are two main types of image sensors on the market: charge-coupled device (CCDs) and complementary metal oxide semiconductor (CMOS) image sensors (CISs). They are different in their manufacturing processes and readout structures, resulting in performance and application differences. CCDs rely on specialized shift registers for data readout, offering low noise and high signal-to-noise ratios, suitable for high-precision measurements. CMOS sensors are compatible with standard CMOS IC processes, allowing the integration of the image sensor, photodetector, and readout circuit on a single silicon chip, supporting cost-effective and miniaturized designs.

**Figure 13:**
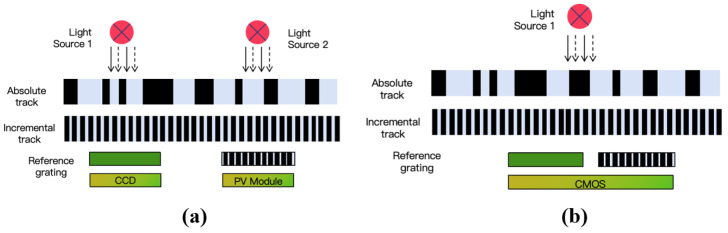
Application of CCD and CMOS image sensors in absolute linear encoders. (**a**) CCD in absolute linear encoder. (**b**) CMOS image sensor in absolute linear encoder.

Both CCD and CMOS sensors can be used in absolute linear encoders, as shown in Figure 13. In CCD-based absolute linear encoders, the CCD captures images of the absolute code channels, while photodiode arrays receive Moiré fringes. Due to spatial constraints and limited light spot sizes from a single LED, the system in Figure 13a uses two LEDs. In CMOS-based absolute linear encoders, the advantages of CMOS integration allow the image sensor, photodiode array, and readout circuit to be combined on a single chip, enabling single-source illumination and reducing the system size, as shown in Figure 13b.

**Figure 14:**
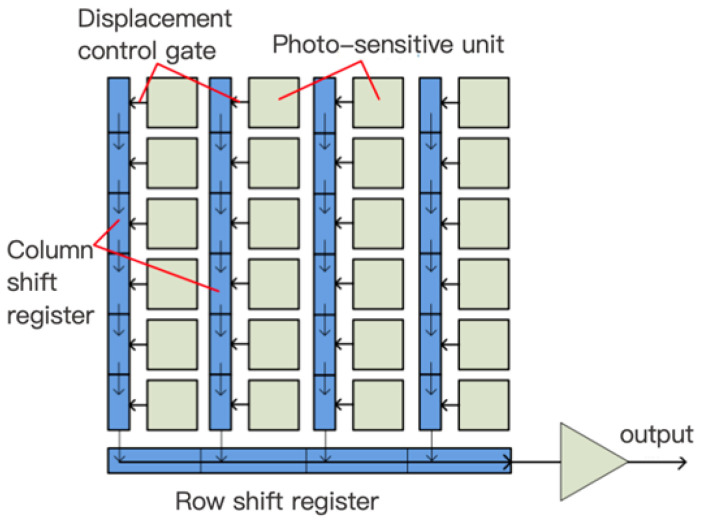
Line-interleaved frame transfer CCD system structure.

Figure 14 shows a typical line-interleaved CCD system. Each pixel column contains a shift register, separated from the pixel by a transfer gate. During exposure, a photogenerated charge accumulates in the pixel potential well, while the transfer gate is low, isolating the well from the shift register. At the end of exposure, the gate switches to high, transferring the charges to the register, after which the gate returns to low. The shift register, driven by a clock, outputs the accumulated charges.

For CMOS systems, image analysis algorithms based on deep learning are widely used in identifying stripe lines and calculating positions due to their high efficiency and accuracy. For line-scan cameras, large-pixel CMOS sensors combined with high-sensitivity area gratings can achieve sub-pixel-level linear displacement calculation. Grating images typically have regular periodic structures, allowing displacement computation using pixel coding techniques, such as precise template matching or conventional correlation analysis. For higher resolutions, sub-pixel interpolation and correlation-based localization algorithms are introduced, offering two advantages:

(1) Strong robustness against illumination variations and noise;
(2) High efficiency and real-time computation capabilities.

Therefore, digital image correlation (DIC) technology is widely used in displacement and deformation measurement, showing good adaptability even in harsh manufacturing environments [135,136]. It compares images of the same object before and after motion or deformation, using sub-pixel interpolation [137] to precisely calculate the displacement. Traditional DIC is mainly applied to surface deformation analysis [138], with limitations in large displacement measurement. To overcome the shortcomings of 2D area scan cameras, line-scan cameras with large-format sensors are ideal for large displacement measurement. These cameras generate single-line pixel images using CMOS or CCD arrays and complete acquisition under high speeds, high spatial resolutions, and relatively simple illumination. Compared with 2D cameras, line-scan cameras offer higher efficiency and precision in one-dimensional high-accuracy measurement [139].

In terms of improving line-scan cameras’ accuracy, sub-pixel image alignment is critical. The most common method is cross-correlation peak search between two aligned images [140]. Cross-correlation can be implemented in both the spatial and Fourier domains, maintaining high registration accuracy and robustness under background light variations and noise [141,142].

Spatial domain methods typically compute least-squares errors [143,144] or direct cross-correlations [145,146] between the reference and sensed images.

Fourier domain methods are more efficient in scenarios with computational speed constraints or frequency-related noise [147,148]. A typical Fourier domain method embeds the cross-power spectrum into a zero-padded matrix, performs an inverse FFT to obtain the upsampled phase correlation, and locates the peak to determine sub-pixel translation [149].

To improve the precision and efficiency, various enhancements have been proposed. For example, interpolation-based iterative intensity interpolation [142] and correlation interpolation [150] improve peak localization but are sensitive to noise [151] and dependent on the interpolation quality [152]. Foroosh et al. [141] proposed using Sinc functions to approximate Dirac δ functions in the frequency domain, reducing the computational complexity, with a resolution of 0.05 pixels. Other studies have applied phase-shifting algorithms to pseudo-periodic images for sub-pixel performance, limited to <200 μm. Vergara et al. [153] used digital holography for long-working-distance planar position measurement, achieving a 50 nm resolution. Guizar-Sicairos et al. [154] proposed the single-step discrete Fourier transform (SSDFT) method, reducing the computation time and memory while maintaining FFT-level precision, validated in various machine vision applications [155,156,157].

Overall, image processing-based linear displacement measurement can achieve high accuracy and resolutions, but limitations remain. Some research cannot simultaneously satisfy the needs for high precision and long ranges, system integration is low, special circuits are needed for noise suppression, and research on contamination and interference resistance is limited. In the future, image processing-based grating linear displacement measurement systems are expected to evolve toward high flexibility, robustness, and fault tolerance, enabling high-accuracy, high-resolution, long-range linear displacement measurement.

### 2.2. Encoding and Decoding of Linear Encoders

The key to absolute position encoders lies in the coordinated design of encoding techniques and decoding algorithms. Encoding refers to engraving structural information on the linear encoder that carries a unique positional identifier, typically realized through transmissive/opaque or reflective/non-reflective patterns. Decoding is performed by capturing the grating line images with CCD or CMOS detectors and analyzing them via image processing algorithms to obtain high-precision absolute displacement [158].

#### 2.2.1. Traditional Absolute Encoding Methods

In absolute linear encoders, specific optical patterns are engraved on code tracks, and photodetectors read them to directly obtain the current absolute position without requiring a reference zero. The core principle is the design of the grating line distribution and code track structure according to different encoding methods.

Natural binary encoding divides the linear encoder into 2^*n*^ positions, each corresponding to a unique *n*-bit binary code. High and low bits are distributed across different code tracks (or in linear sequence), and the photodetector directly outputs the corresponding binary value for absolute positioning. This method is structurally simple but may produce transient errors when multiple bits change simultaneously during position jumps.

Gray code ensures that only one bit changes between adjacent positions, reducing the risk of misreading during transitions [159]. This is particularly suitable for linear encoders in environments with mechanical vibration or sampling timing errors. The captured Gray code must be converted to binary using the formula

(11) $$G_{i}=B_{i}⊕B_{i+1}\;\left(n-1\geq i\geq 0\right)$$

where each bit of the binary code is XORed with its logically right-shifted value, while the most significant bit remains unchanged. Increasing the measurement range requires additional code tracks.

The conversion from binary to Gray code is as follows: starting from the least significant bit, XOR each bit with the bit to its immediate left to generate the corresponding Gray code, keeping the highest bit unchanged. In short, XORing a binary code with its right-shifted version produces the Gray code.

Matrix encoding places multiple concentric tracks of different resolutions (e.g., 2-track, 8-track, 2048-track) on a code disk. Light passing through slits is controlled such that the high/low outputs from photodetectors uniquely correspond to the absolute angular position [160]. This method is common in rotary absolute linear encoders and enables large-range absolute positioning with a limited number of tracks.

Displacement continuous encoding generates non-repeating code sets via a “left shift + complement” strategy, with n−1 bits unchanged between adjacent codes [161]. This improves signal continuity and interference resistance, suitable for high-reliability applications.

Pseudorandom codes are sequences of binary “0”s and “1”s that appear random but are deterministic. Longer sequences have longer periods, with independent bit values and equal occurrence of symbols. Advantages include the ability to read the absolute position at any time; disadvantages include lower contamination resistance and reliability.

Common m-sequences are generated by linear feedback shift registers (LFSRs). The register is loaded with an initial value, and, with each clock pulse, the contents shift right, and a new bit generated according to the feedback rule is written into the first stage, forming a maximum-length binary sequence [158].

Recent studies have also explored nonlinear optical effects for encoder signal generation. Astuti et al. proposed a second-harmonic-wave angle sensor using a femtosecond laser beam, which demonstrated high sensitivity and a compact design for displacement and angle metrology [162]. This indicates that nonlinear optics could serve as a promising foundation for next-generation absolute encoding schemes.

In absolute linear encoders, each pseudorandom code bit corresponds to a grating line, with “1” representing a bright stripe and “0” a dark stripe. Photodetectors (e.g., linear CCDs) capture the stripe signal, compare it with a mask template and code table, and uniquely decode the current position, enabling direct positioning without a reference zero [159]. Compared with true random codes, pseudorandom codes are controllable, have sufficiently long periods, and are easy to manufacture and replicate, making them suitable for absolute linear encoders.

**Figure 15:**
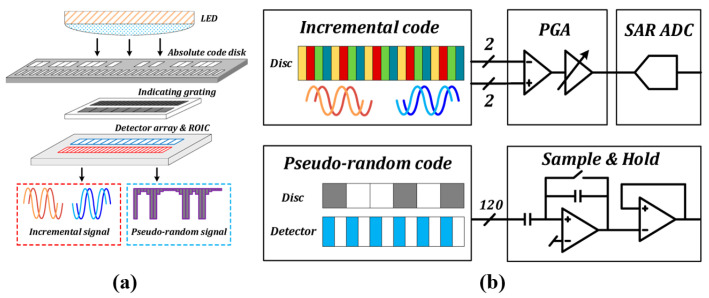
Pseudorandom coded linear encoder. (**a**) Principle of angular displacement reading. (**b**) Mixed-channel optical encoder chip architecture.

As shown in Figure 15, fine tracks use incremental coding, and coarse tracks use pseudorandom coding. Hybrid encoding combines the advantages of incremental and absolute encoding. In the pseudorandom code channel, white and gray areas represent different shading states.

Through these encoding methods, absolute linear encoders can output a unique position immediately upon power-on, eliminating the need for homing and significantly improving the measurement efficiency and system response. The readhead internally performs preliminary decoding from optical stripes to digital codes, generating position data that can be transmitted directly via serial protocols (e.g., EnDat, SSI, BiSS), providing a reliable foundation for subsequent absolute decoding and data verification.

In addition, the optical information processing of traditional absolute linear encoders mostly relies on linear optical systems. Although such systems possess advantages including structural stability and controllable costs, they have limitations in terms of nonlinear information encoding and the improvement of the measurement accuracy in complex scenarios. In recent years, research aimed at realizing nonlinear encoding based on linear optical materials has provided a new direction for the optimization of optical systems in linear encoders. Li et al. [163] systematically analyzed the performance of different nonlinear information encoding strategies in diffractive optical processors based on linear optical materials, proposed two core schemes—phase encoding and data repetition encoding—and verified the role of nonlinear encoding in enhancing the information processing capabilities of optical systems through the MNIST, Fashion-MNIST, and CIFAR-10 datasets.

#### 2.2.2. Serial Communication Protocols

**Figure 16:**
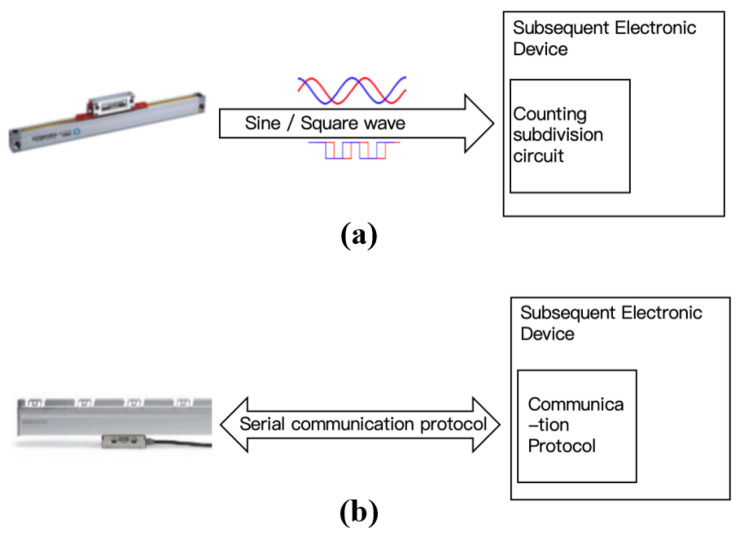
Linear encoder position information transmission. (**a**) Incremental scale transmission. (Red and blue lines denote two sinusoidal interference signals with a 90° phase shift.) (**b**) Absolute scale transmission.

Absolute and incremental linear encoders differ not only in how they obtain position information but also in how this information is transmitted. As shown in Figure 16, incremental encoders directly output Moiré signals to the downstream electronics, which then compute the position through counting and interpolation. In contrast, absolute linear encoders calculate the position internally within the readhead and communicate this information with the downstream electronics via a bidirectional serial communication protocol.

Bidirectional serial communication protocols not only transmit position information but can also convey the encoder’s operating status and manufacturing data and provide a register space for downstream devices to write parameters such as the encoder ID. In short, the communication protocol acts as the “language” between the absolute encoder and the controller, allowing the encoder to interface with machine controllers (CNC, PLC, drives, etc.).

There is no single unified standard: some are defined by CNC manufacturers (e.g., FANUC Serial Interface, Mitsubishi High Speed Serial Interface), some by encoder manufacturers (e.g., EnDat, CPE-Bus), and some by digital signal processing standards (e.g., SSI). BiSS was developed by IC-Haus, a sensor manufacturer. Many encoder manufacturers support multiple protocols to promote compatibility. For example, HEIDENHAIN offers protocol conversion modules to translate EnDat into FANUC or Mitsubishi protocols, simplifying integration with CNC systems. In China, CNC systems largely adopt foreign encoder protocols due to historical and technological reasons. While a domestic standard (CPE-Bus) was introduced in 2012, it has seen limited adoption due to the performance gap with foreign encoders.

**Figure 17:**
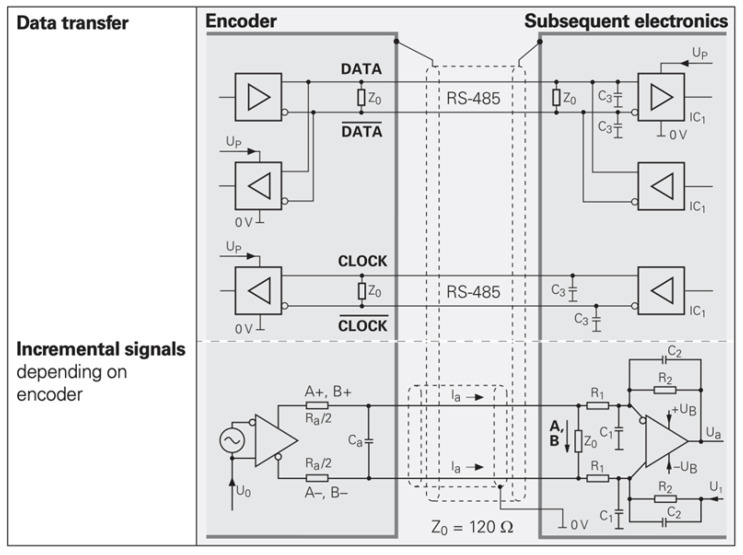
EnDat protocol hardware interface.

EnDat is a proprietary protocol developed by HEIDENHAIN, supporting position readout (angular or linear), register read/write, and encoder status monitoring. As shown in Figure 17, the interface uses RS-485 signaling, with a unidirectional clock line provided by the controller for synchronization, as well as a bidirectional data line whose direction is determined by the command sent.

**Figure 18:**
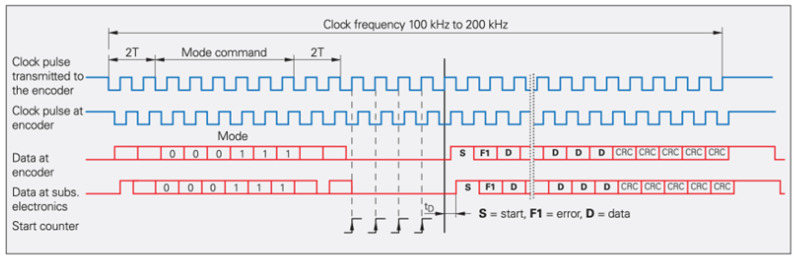
EnDat protocol timing diagram.

For example, with command “000111” (Figure 18), the controller initially occupies the data line and sends clock pulses. On the first falling edge, the encoder latches the most recent position. The command is then transmitted, and the encoder reads it on the third rising edge. After sending the command, two dummy pulses allow the data line direction to switch, and then the encoder transmits position data followed by a CRC check code.

**Figure 19:**
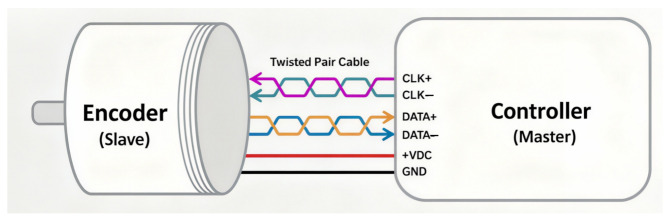
Master–slave relationship between encoder and controller in SSI protocol.

Synchronous Serial Interface (SSI) is a widely used point-to-point serial communication protocol. Its simplicity and long history make it popular in industrial applications. For absolute linear encoders, the SSI provided by EPC is the simplest communication protocol. SSI operates in a master–slave configuration: the controller acts as the master, and the encoder acts as the slave. The master controls the data transmission speed via its clock frequency, and each clock pulse from the master triggers the slave to respond with one bit of information until the entire data packet is received. Figure 19 illustrates this master–slave relationship.

**Figure 20:**
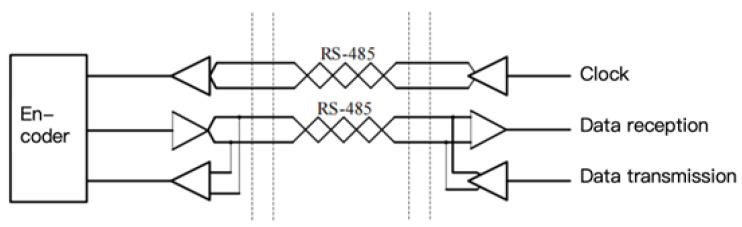
Hardware interface of the SSI communication protocol.

FAGOR’s absolute linear encoders use the SSI protocol, and their hardware architecture is shown in Figure 20. Similarly to the EnDat protocol, SSI uses RS-485 signaling, with a unidirectional clock line and a bidirectional data line.

**Figure 21:**
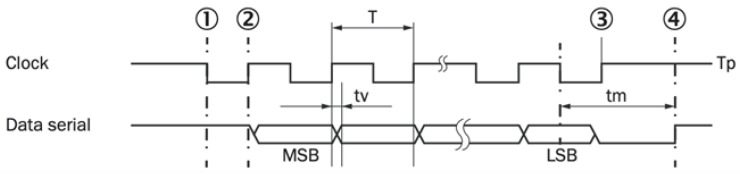
Pulse diagram of data transmission for the SSI communication protocol. (Step 1: Start of data transmission; Step 2: Data valid time (tv) after clock edge; Step 3: End of data word (LSB); Step 4: Pause time (Tp) before next transmission.)

In control systems, SSI typically transmits only position information, while parameter configuration must be performed separately on a PC. Consequently, SSI is less flexible than EnDat but much simpler in operation. As shown in Figure 21, the downstream device sends clock pulses to the encoder, which then returns the position information (angular position for rotary encoders or linear position for linear scales) synchronized to the rising edge of each clock pulse.

**Figure 22:**
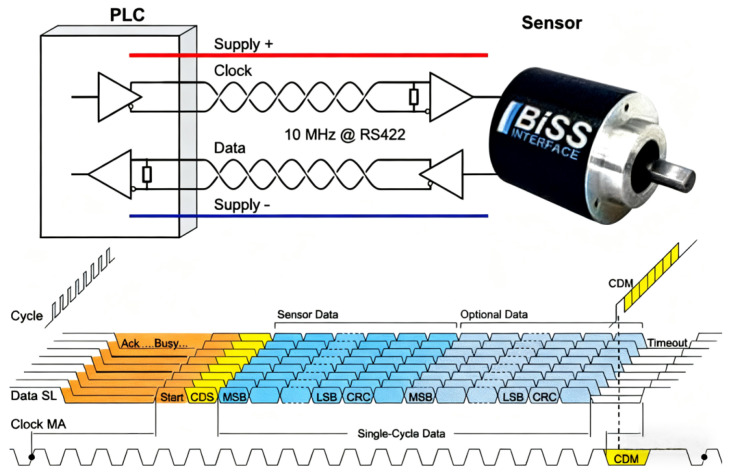
Typical BiSS protocol encoder connection.

The BiSS communication protocol is an open serial communication protocol proposed by the German company IC-Haus. The BiSS protocol is divided into Sensor Mode and Register Mode. Sensor Mode is used for transmitting position or angle information, while Register Mode is used for transmitting parameters. As shown in Figure 22, the BiSS protocol supports one-to-one or one-to-many communication; in practical applications, one-to-one transmission is more commonly used.

CPE-Bus is an encoder communication bus defined in China’s standard JB/T 11505–2013 [164]: Displacement Encoders CPE-Bus Bidirectional Serial Communication Protocol. It was developed under the supervision of the China National Standardization Technical Committee for Measuring Instruments. It is designed for bidirectional serial communication in both linear and rotary displacement encoders.

The protocol uses a strict layered design, comprising a physical layer and a protocol layer, to standardize the terminology, abbreviations, interface structures, data frame formats, communication timing, synchronization mechanisms, and error detection and safety measures. At the physical layer, CPE-Bus typically employs RS-485 or similar noise-resistant bus structures, supporting long-distance data transmission and enhancing system reliability. The protocol layer defines the frame structures—including start bits, data bits, CRC checks, and stop bits—along with the handshake logic, ensuring efficient data exchange between the encoder and controller. The CRC mechanism detects and corrects transmission errors, improving the communication safety and stability.

CPE-Bus is designed to be functionally extensible, user-friendly, and highly reliable, aiming to establish a unified communication standard for domestic displacement encoder systems and facilitate interoperability between encoders and control devices from different manufacturers.

Compatibility is a critical factor in the design and application of absolute linear encoders, as these devices are often integrated into complex systems that involve motion controllers, industrial communication buses, and various hardware interfaces. From the perspective of hardware interfaces, modern encoders are required to support standardized protocols such as SSI and EnDat, which allows seamless data exchange with controllers and reduces system integration costs [165,166]. Furthermore, the implementation of protocols such as EnDat2.2 not only improves the data transmission efficiency but also improves system reliability and synchronization in high-precision positioning applications [166].

On the software and communication side, compatibility ensures interoperability between encoders and industrial networks such as EtherCAT, PROFINET, and CANopen. This guarantees that encoders can be deployed across heterogeneous automation platforms without requiring extensive adaptation. In addition, compatibility with safety standards (for example, functional safety protocols integrated in EnDat) has become increasingly important in robotics and aerospace applications, where both redundancy and fault tolerance are essential [167].

Overall, ensuring compatibility not only improves flexibility in system integration but also extends the applicability of absolute encoders in diverse industrial scenarios. As a result, trends in encoder design emphasize standardized interfaces, backward compatibility, and support for next-generation communication protocols.

#### 2.2.3. Absolute Decoding

Although the measurement accuracy of absolute linear encoders is influenced by a combination of internal and external factors, the ultimate determinant is the precision of code track localization and decoding [168]. Currently, two main methods are employed for absolute position decoding: electronic subdivision and image-based decoding.

Electronic subdivision obtains a low-resolution absolute position by reading the current code track, while combining the subdivision signals of the periodic gratings to achieve high-resolution information [169]. This approach generally requires both absolute and incremental code tracks. During measurement, the absolute code track is captured by image sensors such as CCDs and converted into square-wave signals. The signals are then decoded by analyzing their width and amplitude, translating pseudorandom codes into the current absolute position. Incremental code tracks typically use periodic gratings, with Moiré fringes generated by the relative motion between the reference and scale gratings. These fringes are converted into sinusoidal signals by photodetector arrays and further subdivided for fine positional resolution [170,171,172].

However, this method requires dual-read devices, significantly increasing the system cost. Moreover, sinusoidal signals are sensitive to electromagnetic interference and environmental noise, making electronic subdivision systems both expensive and demanding in terms of anti-interference performance [173]. Advanced approaches, such as the zero-phase bandpass filter Sin-Cos encoder subdivision proposed by Haoning Zhao et al. (2019), enable high-precision electronic subdivision suitable for high-speed encoder applications [174]. Yang et al. (2023) introduced a single-pass absolute linear encoder design combining a bottom absolute track with a top periodic track, using Moiré subdivision to achieve high-resolution measurement while maintaining a compact structure and resistance to contamination [175].

Image-based decoding, in contrast, does not require incremental tracks. It directly identifies and decodes the position based on the absolute code track images, simultaneously providing both coarse and fine positional information. For example, Wang et al. [176] extracted grating edges using grayscale analysis and image subdivision to measure displacement; Li Yanfeng et al. [177] applied virtual instrumentation combined with image processing algorithms to precisely extract grating displacement data; Cai et al. [178] used empirical mode decomposition (EMD) to identify edge trends and determine thresholds adaptively; Yu et al. [179] developed edge extraction algorithms based on quadratic fitting and multi-line fusion to decode the code width; and Yu [180] further combined image centroid calculation with linear fitting and ratio methods for high-precision subdivision. To enhance noise immunity and code track recognition stability, Yu et al. [181] also proposed multi-line fusion with n-th-order denoising algorithms. While image-based decoding reduces the hardware complexity and cost, it demands high image quality, and algorithm parameters are sensitive to environmental changes, meaning that adaptability and robustness require targeted optimization in practical applications. Kim et al. [182] designed a three-step operation of “pixel magnification–scalar multiplication with weight matrix–image superposition” for the features where the output dimension is greater than the input dimension in the decoding stage. Specifically, each pixel of the low-dimensional encoded input (e.g., the compressed position signal of a linear encoder) is first magnified. Subsequently, the magnified pixels are projected onto the weight matrix loaded on the LCD (a pre-trained decoding template, organized in column vectors rather than the row vectors used for encoding) to perform scalar multiplication. Finally, through image superposition, the high-dimensional absolute displacement information is directly outputted.

A study [183] proposed a precision positioning system based on image grating technology. Compared with traditional optical encoders, this system offers improved robustness and flexibility for one-dimensional positioning. It employs an image grating attached to a linear stage as the target feature, as well as a line-scan camera as a fixed displacement reader. By measuring the positions of specific features in the image and applying sub-pixel image registration methods, the system can determine the stage position. Computational efficiency is enhanced through frequency-domain pattern correlation and sub-pixel registration, while lens distortion compensation improves the measurement accuracy. Experimental results demonstrate that the developed image grating system achieves ±0.3 μm precision over a 50 mm range and ±0.2 μm over 25 mm. By using different optical components, the system can be tailored in terms of working distance, measurement range, and resolution to suit various precision measurement applications.

Furthermore, the decoding process of an absolute linear encoder essentially involves the accurate retrieval of displacement information from optical measurement signals. Its core challenges lie in addressing noise interference in encoded signals, nonlinear distortion, and the collaborative decoding of multiple code tracks. Within the field of computational optics, the collaborative optimization framework of “optical encoding–computational decoding” provides crucial theoretical and methodological support for innovations in absolute decoding technology. In 2025, Liheng Bian et al. proposed that an end-to-end joint optimization framework [184], by deeply integrating the optical encoding process (e.g., modulation matrix) with decoding networks (e.g., CNN, RNN), can leverage backpropagation algorithms to synchronously optimize the encoding parameters and decoding models, thereby significantly enhancing the accuracy of information retrieval. This approach can be directly applied to the design of absolute decoding systems.

Additionally, solutions for the “physical twin gap” in computational optics hold great significance for improving the robustness of absolute decoding. Liheng Bian et al. further noted that there exist discrepancies in bit depth and numerical range between optimized digital encoding parameters and actual optical components (e.g., modulators), which necessitate adaptation through strategies such as binarization algorithms and matrix decomposition [184]. In the context of absolute linear encoders, this issue manifests specifically as “deviations between theoretical encoding patterns and actual grating rulings” and “discrepancies between optical signal noise and ideal encoded signals”. The decoding process can be optimized through similar methods: introducing physical constraints (e.g., ruling error models, noise statistical characteristics) into the training of decoding networks to enable the decoding models to adapt to practical hardware deviations.

### 2.3. Performance Indicators of Absolute Measurement

Absolute optical encoders play a crucial role in high-precision position measurement. As shown in Table 1, their key performance indicators include the resolution, accuracy, measuring range, and response speed. In recent years, optical encoders have continuously evolved toward higher precision, higher resolutions, longer measuring ranges, faster responses, and improved environmental adaptability and reliability [95].

In terms of high precision and a high resolution, HEIDENHAIN (Germany) has developed optical encoders with a grating pitch of 0.512 μm using interferometric scanning principles. These products achieve a resolution of up to 1 nm and accuracy of ±0.5 μm and are widely applied in ultra-precision measurement scenarios such as grating fabrication, semiconductor manufacturing, and piezoelectric ceramic nano-positioning platforms. Regarding the measuring range and response speed, companies such as HEIDENHAIN, RSF, and Renishaw have developed steel-band reflective optical encoders capable of measuring up to 30 m and supporting maximum motion speeds of 480 m/min, suitable for large-scale equipment or long-stroke measurement needs. For reliability, mainstream manufacturers commonly adopt single-field scanning techniques and anti-contamination designs, significantly enhancing interference immunity and long-term stability.

#### 2.3.1. Measuring Range and Stroke

The measuring range refers to the selectable minimum and maximum measurement lengths among a series of products, while the stroke indicates the maximum measurable length of a single optical encoder. Because manufacturing high-precision encoders is extremely demanding, increasing the length often results in significantly higher costs. Therefore, in practical applications, the stroke should be selected according to specific measurement requirements to balance performance and cost.

#### 2.3.2. Measurement Accuracy

One of the foremost challenges of the position sensing is to achieve high precision over a large measurement range [185]. The accuracy of an optical encoder primarily depends on the quality of the grating lines and the scanning process, followed by the performance of subsequent signal processing and subdivision circuits, as well as guiding errors between the index grating and the scale grating. The measurement accuracy can be evaluated in terms of two aspects: the positional error over the entire measurement range and the positional error within a single signal period.

For the measurement range, there are two common ways to define accuracy. The first defines the maximum error *F* within any 1 m of the measurement range as ±*a*, with ±*a* being the accuracy grade, independent of the total measurement length. The second defines accuracy as ±(*a* + 2*L*/1000) μm, where *a* is a constant and *L* is the measurement range, a definition mainly used by MITUTOYO (Kawasaki City, Kanagawa Prefecture, Japan).

The positional error within a single signal period also affects the accuracy. It relates to the signal period, grating quality, and scanning quality. Typically, it is ±*a*% of the signal period. For example, HEIDENHAIN’s open-type encoders have a single-period positional error of approximately ±1%.

#### 2.3.3. Grating Pitch and Resolution

In an optical encoder measurement system with a high-precision grating as the core, the grating pitch is the measurement benchmark [186]. The grating pitch refers to the distance between two physical lines on the grating. Before signal subdivision or multiplication, moving one grating pitch corresponds to one output signal cycle. To achieve higher measurement precision, two approaches can be used: reducing the grating pitch or applying signal multiplication techniques. Multiplication densifies the original signal, effectively shortening the period of the sine wave and reducing the corresponding measurement distance per cycle, thereby improving the measurement quality.

The resolution is defined as the smallest measurable step after applying signal multiplication to the grating pitch [187]. Due to limitations in grating manufacturing, measurement accuracy, and signal processing circuits, the degree of signal multiplication is finite [188]. Therefore, manufacturers recommend a specific measuring step (i.e., resolution) for each encoder, ensuring accurate measurements at the encoder’s nominal accuracy [189].

#### 2.3.4. Motion Speed

The motion speed of an optical encoder is the maximum movement speed that it can support while maintaining signal quality and measurement accuracy, usually expressed in m/s. This indicator directly relates to the encoder’s suitability for high-speed applications, such as CNC machines and high-speed inspection equipment.

Factors affecting the motion speed include the scanning response speed of the photoelectric signal, the bandwidth of the signal processing circuits, and the response capabilities of the encoder. If the speed is too high and the signal bandwidth is insufficient, signal distortion may occur, leading to position signal loss or accuracy degradation. Studies have shown that the scanning speed and subdivisional error (SDE) increase linearly. For instance, Gurauskis et al. [190] reported that, as the scanning speed increases, the SDE also rises, manifested as the distortion of the interference signal shape and reduced measurement accuracy.

### 2.4. Selection of Absolute Linear Encoders

Absolute linear encoders represent critical components for high-precision displacement measurement in modern industry, scientific research, and advanced manufacturing. Given that application scenarios differ in terms of accuracy requirements, operational speed, installation constraints, environmental conditions, and compatibility with control system interface protocols, it is imperative to establish a systematic and comprehensive framework for encoder selection. Such a framework constitutes a key prerequisite for ensuring efficient and reliable system performance. The selection of absolute linear encoders should consider the following aspects.

(1) Encoding Method: The choice between quasi-absolute linear encoders and absolute encoding schemes is application-dependent. Quasi-absolute linear encoders require a homing operation after power interruption to re-establish the absolute position. Despite this limitation, they can provide rapid relative position feedback during operation at a comparatively low cost, making them a practical and economical solution for general-purpose CNC lathes and other machine tools with moderate accuracy demands where homing is acceptable. For applications requiring high integration within limited space, embedded quasi-absolute linear encoders offer additional advantages. Absolute linear encoders, by contrast, provide continuous absolute position data without the need for homing, thereby improving the startup efficiency and enhancing the positioning accuracy. They are widely adopted in automated production lines and precision machining centers, where stringent requirements on productivity and accuracy prevail. Conventional multi-track encoding remains a mature solution, utilizing multiple parallel tracks to resolve absolute positions. This approach is well established in engineering practice and includes coding schemes such as natural binary, Gray code, vernier code, and hybrid absolute–incremental codes. Nevertheless, the need for multiple tracks and sensors increases the system complexity and cost while hindering miniaturization. In contrast, single-track encoding employs aperiodic code sequences—such as pseudorandom codes, continuous displacement codes, or hybrid formats—enabling compact designs with reduced structural complexity. However, challenges remain regarding sequence diversity, real-time decoding efficiency, and long-range stability, which require further technological advancement.
(2) Core Performance Metrics: Measurement accuracy constitutes the principal criterion in encoder selection. Importantly, accuracy levels must be aligned with the actual precision requirements of the machine tool, as the indiscriminate pursuit of ultra-high accuracy often leads to disproportionate cost escalation [1]. For example, equipping a standard CNC machine with an ultra-high-precision encoder not only increases the procurement costs but also demands specialized installation, calibration, and maintenance resources, thereby raising the overall lifecycle costs. Rational selection should thus correspond to the machine’s designated machining accuracy class: for machines with general machining tolerances of ±0.01 mm, encoders offering accuracies of ±(0.001–0.005) mm are typically sufficient. Encoder speed performance is equally critical, as it determines the capacity to provide timely and reliable feedback on axis movement. High-speed machine tools require encoders with a rapid response and high data transmission rates to ensure accuracy under dynamic operating conditions. Furthermore, long-term stability is essential to sustaining measurement accuracy, being largely dependent on the manufacturing precision, material quality, and resistance to external disturbances. Encoders constructed with high-grade materials and refined manufacturing processes exhibit superior resilience against thermal fluctuations, mechanical vibrations, and electromagnetic interference. In demanding industrial environments characterized by significant temperature variations or strong vibration, encoders with enhanced anti-interference design and proven stability should be prioritized to guarantee reliable long-term operation.
(3) Structural Materials: Encoder scale materials and structural designs also play a decisive role in performance. Glass scales offer superior precision and are commonly employed in ultra-precision applications such as optical lens grinding machines. However, they are inherently brittle, typically limited to lengths below 4 m [63], and relatively costly. Their advantages lie in surface flatness and a low coefficient of thermal expansion, which facilitate accurate alignment during installation. By contrast, steel-tape encoders provide greater flexibility and can be manufactured in extended lengths of up to 4 m or more [63], making them more suitable for large-travel machine tools such as gantry machining centers, albeit at the expense of reduced accuracy. Regarding structural design, enclosed encoders integrate the scale within a protective housing, ensuring superior resistance to dust, coolant, and machining debris. In machine tools with sufficient installation space—such as floor-type boring–milling machines—enclosed glass encoders are preferred, as they safeguard the measurement accuracy and prolong the service life. In compact machine tools, however, where installation space is constrained, miniaturized glass or steel-tape encoders provide a more practical solution, enabling flexible integration without interfering with the arrangement of other components, while still ensuring adequate measurement performance.
(4) Communication Protocols: Absolute linear encoders typically transmit position data via serial communication interfaces, with different manufacturers adopting distinct protocol standards. Therefore, protocol compatibility with the numerical control (NC) system is a decisive factor in encoder selection. For instance, HEIDENHAIN encoders frequently employ the EnDat 2.2 protocol, which is noted for its high transmission speed, strong anti-interference capabilities, and seamless integration with HEIDENHAIN NC systems. This makes them particularly suitable for high-precision applications. Encoders from FAGOR commonly utilize the SSI protocol, valued for its universality and robustness and widely compatible with Siemens and other mainstream NC platforms, thereby providing users with versatile integration options. Meanwhile, Renishaw encoders, as well as those developed by the Changchun Institute of Optics, Fine Mechanics, and Physics of the Chinese Academy of Sciences, often adopt the BiSS protocol, which offers high-speed and high-accuracy data transmission. This makes it particularly advantageous in aerospace machining and other fields requiring stringent positional accuracy and real-time feedback.

## 3. Quasi-Absolute Linear Encoder

To balance the demand for high-precision measurement with industrial cost-effectiveness, the quasi-absolute linear encoder has emerged as a compromise solution. Its core concept lies in the coordinated design of an “incremental track + aperiodic auxiliary track”, which preserves the high resolution advantage of incremental systems while using the auxiliary track to provide a coarse absolute position reference, thereby reducing the encoding complexity. Quasi-absolute encoding often relies on the manufacturing processes of embedded microstructures and highly uniform patterns to achieve high resolutions and fault tolerance. In recent years, there have been various advancements in micro–nano processing and large-scale grating fabrication, including the preparation of highly uniform arrays using dielectric film polarization modulation, grating mosaic/splitting strategies, manufacturing techniques for large-scale diffraction gratings, and the interference/Lloyd mirror method for creating variable line spacing etch lines. These process improvements directly support the feasibility and performance enhancement of quasi-absolute encoding [191,192,193,194,195,196]. In terms of the encoding format, quasi-absolute linear encoders can be categorized into non-embedded encoding and embedded encoding, which are introduced in detail below.

### 3.1. Non-Embedded Encoding

In the field of displacement measurement, absolute linear encoders enable the real-time determination of the readhead position through unique position encoding, eliminating the need for a homing operation. However, this technology still faces several challenges [197].

(1) The complexity of encoding and decoding algorithms for absolute tracks increases exponentially with higher precision.
(2) The high-density measurement gratings required for absolute tracks must be fabricated using specialized processes such as electron beam lithography. The micron-level etching precision required results in significantly higher unit manufacturing costs compared to conventional gratings.
(3) Absolute tracks often adopt a multi-track configuration. Clock synchronization issues in the parallel processing of multi-track signals can lead to millisecond-level decoding delays, which are prone to causing cumulative errors in high-speed motion control scenarios.

To balance accuracy and cost-effectiveness, quasi-absolute linear encoders have been introduced to the market. The encoding scale of a quasi-absolute linear encoder incorporates at least two functionally distinct tracks. The primary track is an incremental track, equipped with periodic optical markings that generate regular electrical pulses through relative motion—i.e., incremental signals—enabling high-resolution displacement measurement [48,49]. The auxiliary track features a non-periodic design, where combinations of fixed-length stripes form specific sequences, each uniquely corresponding to an absolute position coordinate within the entire measurement range. The auxiliary track provides a coarse absolute position reference either before or during incremental measurement, allowing for initial or boundary positioning. In design, the auxiliary track may be embedded within the primary track, and thus quasi-absolute encoding can be classified into embedded and non-embedded types.

**Figure 23:**
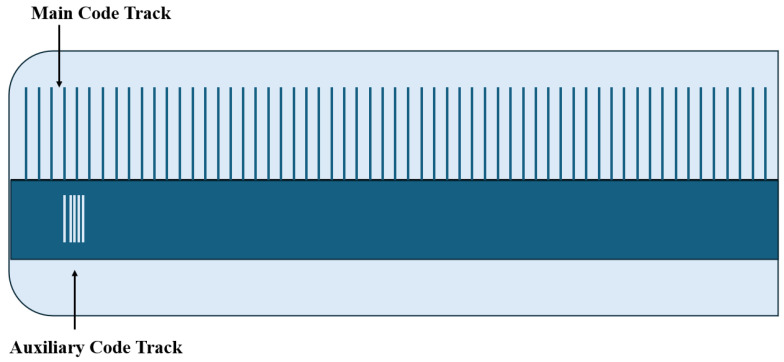
Initial design diagram of quasi-absolute encoding.

The initial design of the incremental code track featured only a single reference mark, which defined an absolute position on the scale precisely corresponding to the start of a signal period. However, this required the system to pass through the reference mark—i.e., to perform a “homing operation”—in order to establish an absolute reference or to recover a previously set reference point. This process could necessitate moving the machine a considerable distance to locate the reference mark [198]. The initial design diagram of quasi-absolute encoding is shown in Figure 23.

To speed up and simplify the homing process, reference marks in the form of distance-coded markings were introduced. These distance-coded reference marks are arranged in pairs, with constant spacing within each pair and varying spacing between pairs.

**Figure 24:**
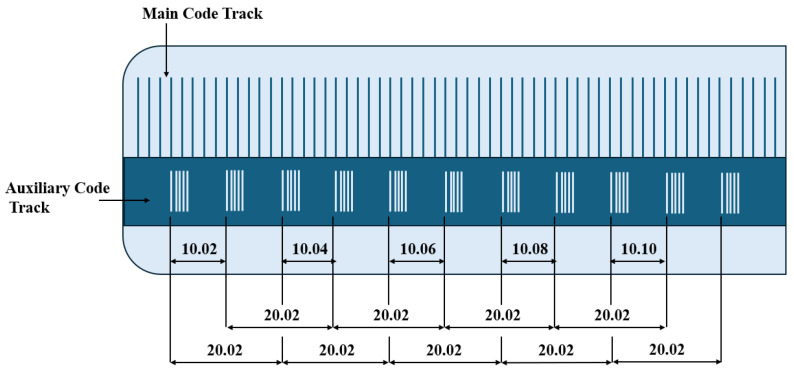
Distance encoding diagram.

On a scale with distance-coded reference marks, the spacing between each pair of adjacent marks is consistent; however, the distance between successive reference marks increases by a fixed increment as the travel distance changes. By reading this distance value, the current absolute position can be determined. When the reading device detects the position code at 10.06 mm, the measurement system can identify the corresponding reference point. Once the system moves beyond the 20 mm distance between reference points, as indicated in Figure 24, the current absolute position can be determined. This design significantly shortens the travel required during the homing process.

**Figure 25:**
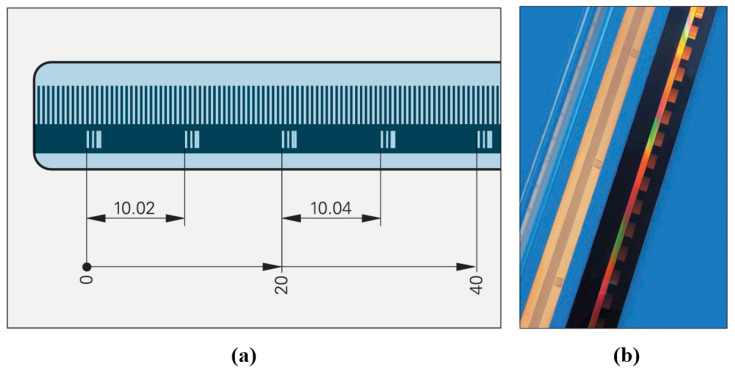
The LIDA 4x3C model of absolute linear encoders. (**a**) Distance Coding Schematic Diagram. (The dark blue represents the opaque part, and the light blue represents the transparent part.) (**b**) LIDA 4x3C model physical image of absolute linear encoder.

The LIDA 4x3C model of absolute linear encoders produced by HEIDENHAIN utilizes this type of distance coding, as shown in Figure 25. Multiple reference marks are inscribed on the auxiliary code track of the linear encoder, arranged at intervals determined by a specific mathematical algorithm. When the corresponding electronic device passes two consecutive reference marks, it only needs to move a few millimeters. By counting the incremental signals between these two marks and applying a specific formula, the absolute reference position can be determined. A long-distance return-to-zero process is not required. The representative product, LIDA 483, features a grating period of 20 µm, a measuring range from 240 mm to 3040 mm, a maximum operating speed of up to 10 m/s, and measurement accuracy of ±1 µm.

The calculation principle of this type of linear encoder is as follows:

(12) $$\begin{array}{cc} P_{1} & =(\mathrm{abs}\;R-\mathrm{sgn}\;R-1)\times N/2+(\mathrm{sgn}\;R-\mathrm{sgn}\;D)\times (\mathrm{abs}\;\mathrm{MRR})/2 \end{array}$$

(13) $$\begin{array}{cc} R & =2\times \mathrm{MRR}-N \end{array}$$

where

$P_{1}$ = Position of first traversed reference mark in signal period;
abs = Absolute value;
sgn = Algebraic sign function (“+1” or “–1”);
MRR = Number of signal periods between traversed reference marks;
*N* = Nominal increment between two fixed reference marks in signal period;
*D* = Direction of traverse (+1 or –1); the rightward traverse of the scanning unit (when properly installed) corresponds to +1.

**Figure 26:**
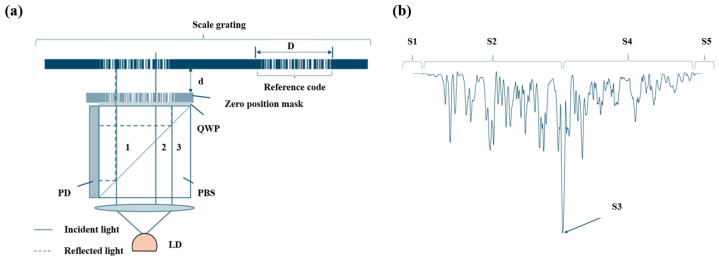
Design of distance coding based on deep learning [199]. (**a**) Working diagram of distance coding. (**b**) Signal generated by distance coding.

Designing and optimizing distance codes with more than 100 bits poses considerable challenges. The research team led by Li from Tsinghua University proposed a distance code design method based on deep learning generative adversarial networks (GANs) while taking diffraction effects into account, as shown in Figure 26. Taking one-dimensional motion as an example, the emitted laser can be decomposed into sub beams. Analyzing the propagation categories of sub beams, it can be found that sub beam types 1, 2, and 3 exist. Among these sub beam types, 1 and 2 pass through the mask, while 3 does not pass through the mask, and its proportion to the overall beam remains unchanged. In addition, S1–S5 represent different overlapping states between the reference code and the mask. S1 and S5 represent non overlapping, S2 and S4 represent partial overlap, and S3 represents complete overlap.

Figure 26a illustrates the working principle of this distance encoding, and Figure 26b shows the signal diagram generated by this distance encoding. For the first time, they utilized deep adversarial networks to generate 150-bit high-precision binary absolute positioning codes. A dataset was constructed to train the model, which included randomly generated codes and their corresponding diffraction propagation performance labels (K/W, where K represents the contrast between the secondary peak and the main peak, and W denotes the full width at half maximum). Finally, verification results demonstrated that the proposed codes can achieve submicron-level positioning accuracy [199,200].

This type of binary distance coding can first be applied in the one-dimensional (*X*-axis) direction and then reused in the *Y*-axis direction to achieve two-dimensional (2D) positioning. The code channel design method eliminates the need for point-by-point processing and only requires drawing short lines, which significantly reduces the micron-level processing cost. With the continuous improvement in requirements for coding accuracy and two-dimensional planar grating fabrication, more stringent technical specifications have been imposed on the manufacturing process for core grating components. Among various grating fabrication technologies, laser interference lithography [201,202,203,204,205,206] has become the mainstream technical route for the large-scale manufacturing of high-density diffraction gratings, as it possesses the advantages of an ultra-high resolution, large-area uniformity, and repeatability simultaneously. In the fabrication of two-dimensional planar gratings, the orthogonal biaxial Lloyd’s mirror [207] is an effective implementation approach.

**Figure 27:**
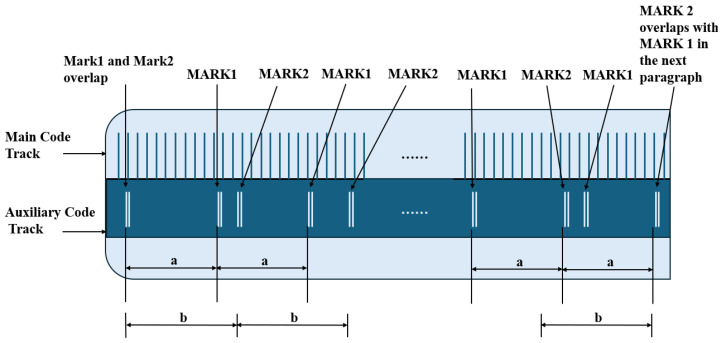
Schematic diagram of multi-segment distance encoding.

In traditional distance-coded reference mark systems, the distance required to determine the absolute position (i.e., position identification resolution) increases with the measuring range of the encoder, resulting in reduced operational efficiency. Based on this, in 2014, Guo Tao from Jilin University proposed a multi-segment distance encoding method, as shown in Figure 27. In this approach, the traditional distance code is treated as a single large segment. A multi-segment structure is formed by varying the spacing between adjacent odd-numbered and even-numbered reference marks across different segments—while keeping the spacing uniform within each segment, the distances differ between segments. In terms of the encoding length, under identical parameter conditions, the novel multi-segment distance code allows for a significantly longer encodable length compared to the traditional distance code. Regarding the position identification resolution, when the encoding length is fixed, the new multi-segment distance code can achieve a higher resolution, enabling more precise position identification [49,208].

In general, quasi-absolute encoding is not absolute in the strict sense. When the readhead is activated or the system is reset, it must first rely on a specific auxiliary code channel to perform an initial position search and locking process—commonly referred to as the “homing operation”. Displacement measurement based on the main incremental track becomes meaningful only after the homing operation has been successfully completed and an accurate absolute position reference point has been established. Ultimately, the actual position of the readhead is determined by accumulating incremental pulses and combining them with the initial reference. Therefore, the absolute positioning capabilities of quasi-absolute linear encoders rely on the successful execution of the homing procedure.

This dependency introduces significant challenges: in the event of a sudden power failure, severe interference causing signal loss, or motion exceeding the system’s position memory capacity—resulting in a measurement interruption—the previously established reference position becomes invalid. The system cannot automatically recover absolute position awareness and must re-execute the entire homing operation to re-establish the reference point. This process significantly extends the system recovery time, increases the operational complexity, and may disrupt critical production or measurement processes, thereby negatively affecting the operational efficiency.

### 3.2. Embedded Encoding

Non-embedded encoding typically employs separate auxiliary and incremental code channels, which are physically isolated from each other. Measurement is performed by independently reading the absolute position information from the auxiliary channel and the displacement information from the incremental channel. However, this separated design can increase the overall size of the linear encoder and may compromise the measurement accuracy due to alignment errors between the channels.

To address the limitations of non-embedded encoding, embedded encoding technology has been developed. In this approach, the auxiliary code channel is embedded within the incremental channel, enabling the integration of both types of information into a single channel structure. This design departs from the traditional separation of code channels by physically superimposing and integrating them, allowing the scale grating to maintain its incremental displacement measurement function while simultaneously providing reference information for absolute positioning.

**Figure 28:**
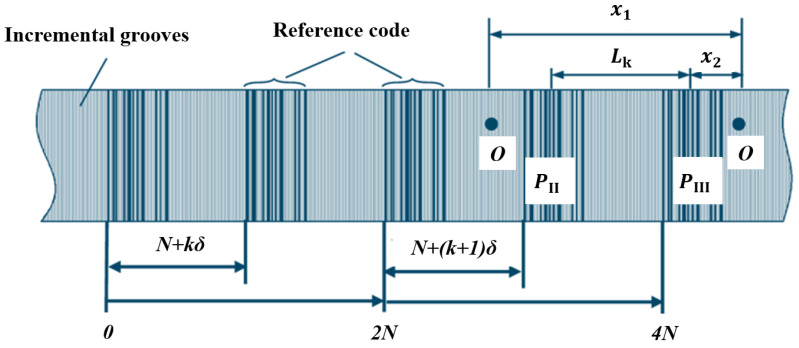
Code domain integration scheme [209].

The linear encoder developed by HEIDENHAIN based on distance encoding technology holds significant reference value in the field of precision measurement and motion control. Building on the design concept and technical framework of this encoder, the research team from Tsinghua University has further proposed an embedded encoding method based on a hybrid positioning approach [209,210,211]. In this method, multiple non-uniformly spaced distance markers are superimposed onto the grating grooves of the incremental code channel. This allows for the acquisition of absolute position information without requiring significant additional space, thereby improving the overall compactness of the system, as illustrated in Figure 28.

**Figure 29:**
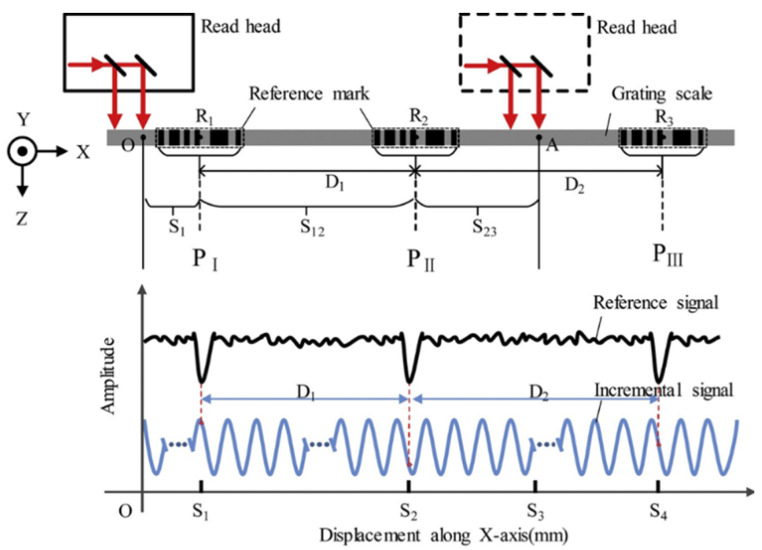
Absolute positioning principles [213].

In the optical path design, the readhead employs a dual-probe configuration, as shown in Figure 29. One probe generates pulse signals through a mask encoded identically to the scale grating markers, enabling the approximate positioning of the markers with accuracy of about 0.5 µm. The other probe, configured for grating interferometry, performs nanometer-scale displacement measurements using 100-subdivision phase information derived from high-quality sinusoidal interference signals. By combining the approximate positioning of the reference pulse signal with the phase information (i.e., phase mark) of the incremental displacement signal within one period, the position within that period whose main phase is closest to the phase mark and contains the approximate position is identified as the precise reference position. This enables positioning repeatability of 10 nm [212].

The hybrid positioning embedded encoding method holds significant academic and application value. First, traditional solutions require additional independent code tracks to obtain absolute positions, which tends to result in an excessive system volume. However, relying on the design of “marker-overlaid incremental code tracks”, this technology achieves absolute position detection without sacrificing space, providing key technical support for the miniaturization and integration development of precision measurement equipment. Second, the dual-probe configuration establishes a synergistic mechanism of “approximate positioning–precision measurement”, balancing macro positioning accuracy and a micro displacement resolution. More crucially, the research team has applied this technology to the design of dual-grating multi-degree-of-freedom absolute grating encoders [214,215,216], further expanding its technical value and application scenarios. The integration of this technology not only meets the requirement for system compactness in multi-degree-of-freedom measurement but also ensures the accuracy of multi-dimensional motion measurement through high-precision positioning repeatability, thus possessing important practical value.

**Figure 30:**
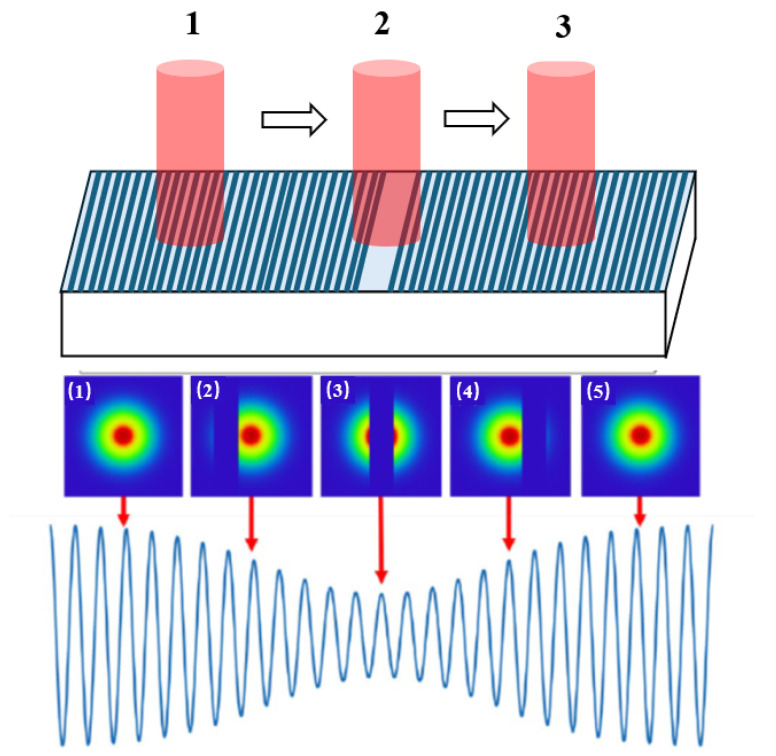
Measurement of light intensity variation as the measuring beam passes through the zero-position marking zone. (The numbers 1–3 and (1)–(5) represent different positions of the Gaussian beam relative to the zero mark.)

In June 2025, Qian Xinge from the Changchun Institute of Optics, Fine Mechanics, and Physics, Chinese Academy of Sciences, proposed an absolute positioning encoding method for grating displacement based on reference-scale fused signal distance encoding, as shown in Figure 30. Specific areas on the surface of the scale grating are processed to remove the reflective coating, forming a zero-position marking zone. The incremental signal is then modulated in intensity, and absolute positioning is achieved by detecting variations in this intensity to encode the distance. This method eliminates the need for complex zero-position marker designs and combines the incremental and reference channels into a single channel, allowing both incremental and absolute measurements to be performed with a single readhead. This facilitates system miniaturization and integration. The absolute positioning accuracy of this method is approximately 0.8 µm [197].

## 4. Absolute Linear Encoder Encoding

Absolute linear encoder encoding does not rely on any external reference points or initial position calibration. Instead, the absolute coordinate of the current position is directly obtained from the encoded pattern on the scale. This “read-and-obtain” feature allows accurate position data to be provided immediately upon power-up, completely eliminating the limitations of quasi-absolute encoding, which requires movement to a reference point to determine the absolute position. It also prevents measurement failures caused by the loss or misidentification of the reference point.

Absolute linear encoders can be classified into two types based on the number of code tracks, namely multi-track and single-track, which are introduced in detail below. At the same time, the optical head and reading structure (such as the optical head with a phase shift reading end, the multi-layer grating interference structure with a near-common optical path), as well as the compact angle/displacement sensor design, have a direct impact on the feasibility and anti-interference ability of the encoding, and they need to be combined with on-machine calibration and error compensation strategies to ensure long-term accuracy and repeatability [217,218,219,220,221,222,223,224].

### 4.1. Multi-Track Absolute Encoding

The multi-track encoding method was the first form of absolute encoding to be developed, including natural binary, Gray code, matrix code, and hybrid absolute–incremental coding, improving measurement reliability [62,225]. Its introduction marked a significant milestone in the evolution of linear encoders from incremental to absolute encoding and it holds considerable importance [169].

This type of linear encoder is marked by multiple code channels that work together to determine the current absolute position. As technology has advanced, multi-channel absolute encoding has developed into various distinctive implementations to balance measurement accuracy, reliability, and the need for device miniaturization. Common methods include natural binary encoding, Gray code, matrix encoding, vernier encoding, and hybrid encoding that combines absolute and incremental code channels [226].

These encoding methods differ in their focus on code channel design, decoding logic, and error control. A detailed introduction to each type of encoding is provided below.

#### 4.1.1. Natural Binary Encoding, Gray Code, and Matrix Encoding

**Figure 31:**
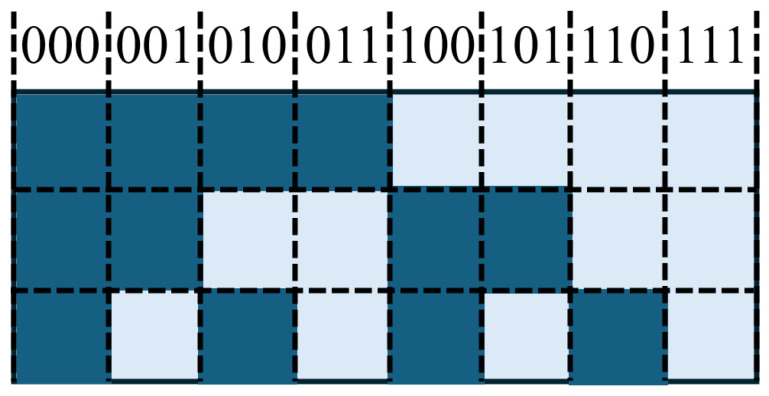
Diagram of 3-bit natural binary code encoding. (Dark blue represents the opaque parts, while light blue represents the transparent parts).

Natural binary code, also known as fixed-weight or non-weighted code, was the earliest encoding method applied in the theory of absolute position encoding. Its core principle is based on bitwise encoding, using only the binary symbols “0” and “1”, with each symbol representing a single encoding bit. In an n-bit encoding system, a complete encoding structure is formed using n code tracks, and the linear encoder is evenly divided into $2^{n}$ equal intervals. Within each interval, the corresponding n-bit binary code is inscribed along the n code tracks. By detecting the code information on these tracks with a photoelectric sensor, the absolute position value of the corresponding interval can be directly determined. The n code tracks are arranged in parallel along the length of the linear encoder, and the number of code tracks exactly matches the number of encoding bits. Figure 31 shows a structural example of three-bit natural binary encoding.

**Figure 32:**
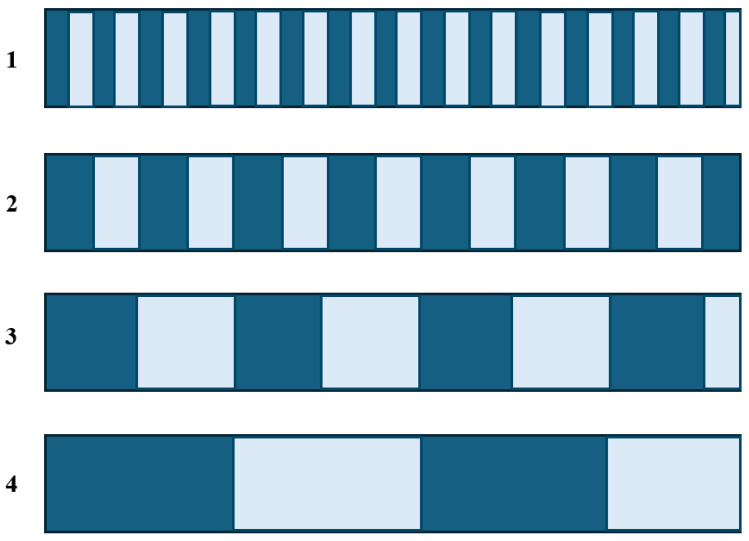
Tracks of the four-track absolute optical encoder. (Dark blue represents the opaque parts, while light blue represents the transparent parts).

In 1994, the German company HEIDENHAIN introduced multi-track absolute position encoding measurement technology. In Figure 32, a segment of a four-track absolute optical linear encoder is depicted, in which natural binary code is used as the encoding method.

**Figure 33:**
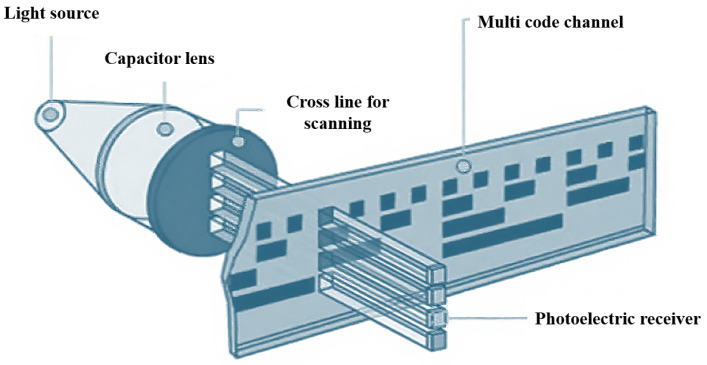
Schematic diagram of the four-track absolute optical encoder.

In this encoding method, multiple code tracks are arranged on the optical linear encoder. Each track is equipped with a photodetector that receives light signals transmitted through the scale from the opposite side. These light signals generate corresponding photoelectric signals, which are then converted from analog to digital and sent to the decoding system. A single-bit position value is extracted from each track, and, by combining the information from all tracks, the complete position information is obtained, as illustrated in Figure 33. By increasing the number of code tracks, the measurement resolution can be enhanced, enabling absolute position detection [50].

In 1997, based on the four-track absolute optical encoder, the German company HEIDENHAIN introduced the LC181 absolute optical encoder with seven code tracks. This device achieved accuracy of ±5 µm or ±3 µm and a measurement resolution ranging from 1 µm to 0.1 µm, following the same underlying principle [227].

This encoding method offers several notable advantages: it provides high encoding efficiency, features intuitive and easily interpretable patterns, adopts simple encoding rules that are easy to implement in engineering applications, and enables the direct reading of binary codes to obtain absolute position information without the need for complex decoding processes. However, the method also presents certain limitations [228].

(1) Improving the position resolution requires increasing the number of code tracks, which in turn necessitates more photoelectric sensors. This added complexity hinders the miniaturization and integration of the device.
(2) If multiple bits change simultaneously between adjacent reading intervals, the photoelectric receiver may generate errors due to asynchronous detection.
(3) During the encoding carry process, multiple code bits often change at the same time. If there are errors in the grating lines, inconsistent carry operations may occur across code tracks, potentially leading to incorrect code readings.

**Figure 34:**
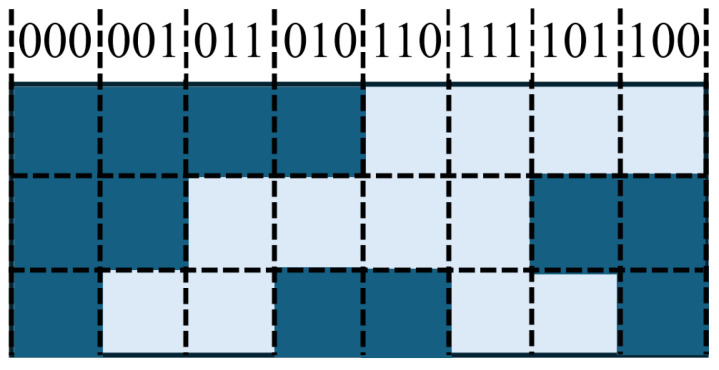
Three-bit Gray code encoding schematic diagram. (Dark blue represents the opaque parts, while light blue represents the transparent parts).

With the advancement of technology, Gray code was first introduced by the renowned German scientist Gray, based on natural binary coding. The digits in Gray code are unweighted and carry no explicit numerical value; hence, it is also referred to as a non-weighted or equal-weight code. Gray code is generated from natural binary code through bitwise exclusive OR (XOR) operations and represents a modified form of binary encoding. A schematic diagram of three-bit Gray code encoding is shown in Figure 34.

The conversion formula for n-bit Gray code is as follows:

(14) $$G_{i}=B_{i}⊕B_{i+1}\;\left(n-1\geq i\geq 0\right)$$

The key advantage of this encoding method is that any two adjacent n-bit codes differ by only one bit, while all other bits remain the same. This minimizes gross reading errors and improves the reliability of natural binary encoding. The disadvantage lies in its encoding format, which resembles that of natural binary code. An n-bit encoding corresponds to n code tracks, requiring an increase in the number of encoding bits to improve the sensor resolution and measurement range, which is unfavorable for miniaturization. During writing and decoding, this encoding method must be converted into natural binary code for reading and cannot be read directly. Nevertheless, the decoding process is straightforward, making it a widely adopted form of absolute encoding [51].

Matrix encoding, as a derivative of Gray code, belongs to the category of non-weighted binary encoding. Its core design concept is to consolidate the multi-turn encoding information of the traditional Gray code into a single code track, thereby reducing the total number of tracks. Specifically, the entire circumference of the encoder is divided into multiple sector intervals, each containing code track information with varying bit lengths. Multiple readheads are deployed to collect electrical signals, and dedicated logic circuits are used to control the gating of each probe at different encoding positions. Ultimately, the output signals from each channel are converted into periodic binary code through matrix logic decoding. A significant advantage of this encoding method is its ability to effectively reduce the number of encoder channels, thereby supporting the miniaturization of devices. However, its application also presents notable limitations: deviations during encoder installation or vibrations during shaft operation can easily cause substantial positional errors between channels, placing stringent demands on the assembly and calibration processes. Moreover, this encoding method requires high performance from the signal processing circuitry [229,230].

#### 4.1.2. Vernier Encoding

**Figure 35:**
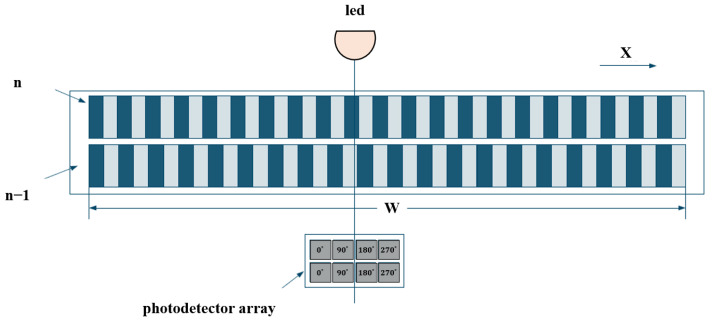
Vernier code absolute optical linear encoder. (Dark blue represents the opaque parts, while light blue represents the transparent parts).

The simplest vernier code absolute optical linear encoder photoelectric receiving unit is shown in Figure 35. The scale grating contains two periodic code tracks. For a maximum measurement length of W, one track has a period of W/n, and the other has a period of W/(n−1), where n is a positive integer. Illumination is provided by an LED, and the signal is received by a 2 × 4 photodiode array. Each photodiode is preceded by an index grating, which generates four-phase Moiré fringes for each of the two code tracks.

**Figure 36:**
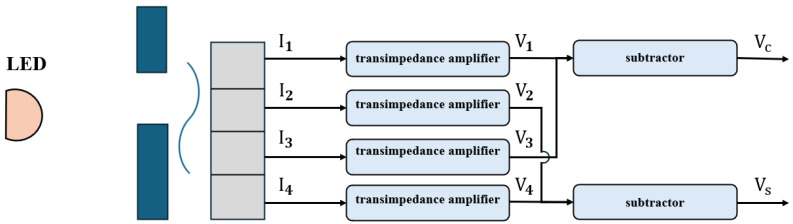
Signal preprocessing circuit of vernier code absolute optical linear encoder.

Figure 36 shows the signal preprocessing circuit of the vernier code absolute optical linear encoder.

Assuming that the common-mode level of the Moiré current signal is half of its peak-to-peak value, the mathematical expressions of the signals shown in Figure 36 can be written as follows:

(15) $$\begin{array}{cc} V_{1} & =R\times I_{0}\left(\cos\left(\theta \right)+1\right) \end{array}$$

(16) $$\begin{array}{cc} V_{2} & =R\times I_{0}\left(\cos\left(\theta +\pi /2\right)+1\right) \end{array}$$

(17) $$\begin{array}{cc} V_{3} & =R\times I_{0}\left(\cos\left(\theta +\pi \right)+1\right) \end{array}$$

(18) $$\begin{array}{cc} V_{4} & =R\times I_{0}\left(\cos\left(\theta +3\pi /2\right)+1\right) \end{array}$$

(19) $$\begin{array}{cc} V_{c} & =V_{1}-V_{3}=2RI_{0}\cos\left(\theta \right) \end{array}$$

(20) $$\begin{array}{cc} V_{s} & =V_{2}-V_{4}=2RI_{0}\sin\left(\theta \right) \end{array}$$

In these equations, θ represents the phase of the Moiré fringe generated by the photodiode corresponding to $I_{1}$, *R* denotes the gain of the transimpedance amplifier, and $I_{0}$ is the common-mode value of the Moiré current signal. Based on these equations, the value of θ can be determined as

(21) $$\theta =\arctan\left(\frac{V_{s}}{V_{c}}\right)$$

Given that Figure 35 contains two encodings, the one with a period of W/n is defined as encoding *a* and the one with a period of W/(n−1) as encoding *b*. The corresponding phase angles $\theta_{a}$ and $\theta_{b}$ can be determined. According to the principle of Moiré fringe formation, the relationship between $\theta_{a}$, $\theta_{b}$, and the displacements $d_{a}$ and $d_{b}$ within a single period of their respective channels is given by

(22) $$\frac{\theta_{a}}{2\pi }=\frac{d_{a}}{W/n}$$

(23) $$\frac{\theta_{b}}{2\pi }=\frac{d_{b}}{W/(n-1)}$$

For an arbitrary position *d*, measurements are taken using both channels, resulting in

(24) $$d=\left(i-1\right)\times \frac{W}{n}+d_{a}=\left(i-1\right)\times \frac{W}{n}+\frac{\theta_{a}}{2\pi }\times \frac{W}{n}$$

(25) $$d=\left(j-1\right)\times \frac{W}{n-1}+d_{b}=\left(j-1\right)\times \frac{W}{n-1}+\frac{\theta_{b}}{2\pi }\times \frac{W}{n-1}$$

where *i* and *j* are positive integers representing the *i*-th and *j*-th periods corresponding to position *d* in the two channels, respectively. Since the two channels differ by only one period over the entire length, the value of i−j can only be 0 or 1. When $\theta_{a}>\theta_{b}$, i−j=0; when $\theta_{a}<\theta_{b}$, i−j=1. If we define

(26) $$\theta_{a-b}^{\prime }=\left\{\begin{array}{cc} \theta_{a}-\theta_{b} & \left(\theta_{a}>\theta_{b}\right)\\ 2\pi +\theta_{a}-\theta_{b} & \left(\theta_{a}<\theta_{b}\right) \end{array}\right.$$

then we obtain

(27) $$\theta_{a-b}^{\prime }=\frac{2\pi (i-1)}{n}+\frac{\theta_{a}}{n}$$

**Figure 37:**
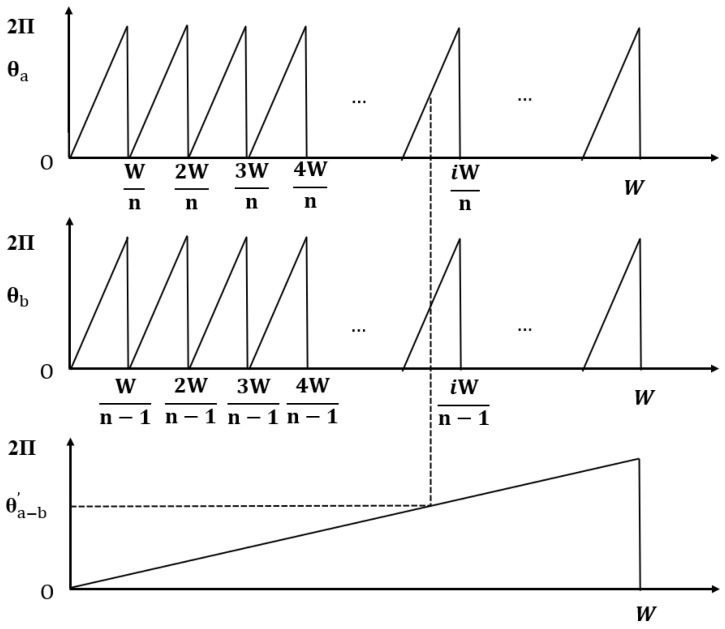
Formation of absolute position in vernier code.

The relationship among $\theta_{a}$, $\theta_{b}$, and $\theta_{a-b}^{\prime }$ can be illustrated using a diagram. As shown in Figure 37, $\theta_{a-b}^{\prime }$ is uniquely determined over the entire measurement length *W*.

**Figure 38:**
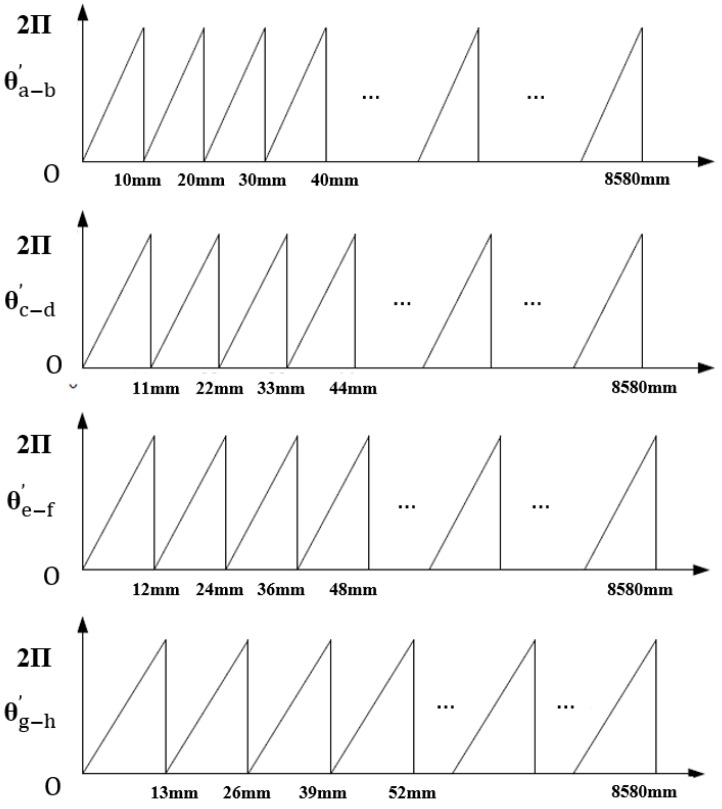
Multi-vernier-code combination for absolute position measurement.

By coordinating multiple sets of vernier codes, a large measurement range (4 m) and high resolution (0.01 µm) can be achieved, along with a certain level of resistance to contamination. Taking the structure shown in Figure 38 as an example, the design employs four sets of vernier codes and eight code channels (labeled a, b, c, d, e, f, g, h). Channels a and b form a vernier code with a 10 mm measurement range for absolute positioning; channels c and d form a code with an 11 mm range; channels e and f form a code with a 12 mm range; and channels g and h form a code with a 13 mm range. By combining the outputs of all eight code channels, an absolute position measurement range of 8.58 m can be achieved. When the parameter n=500 is used, the minimum measurement step size can reach 0.014 µm.

Based on the above analysis, the principle of position measurement in vernier-coded absolute linear encoders is fundamentally derived from the Moiré fringe effect, representing an innovative application of Moiré metrology in engineering measurement. Since absolute position measurement requires the coordinated operation of multiple periodic code tracks, the illuminated area and the detector’s receiving area in this structure are relatively large. This places specific demands on the product’s core performance, such as the maximum operating speed and acceleration, as well as on assembly precision [228].

**Figure 39:**
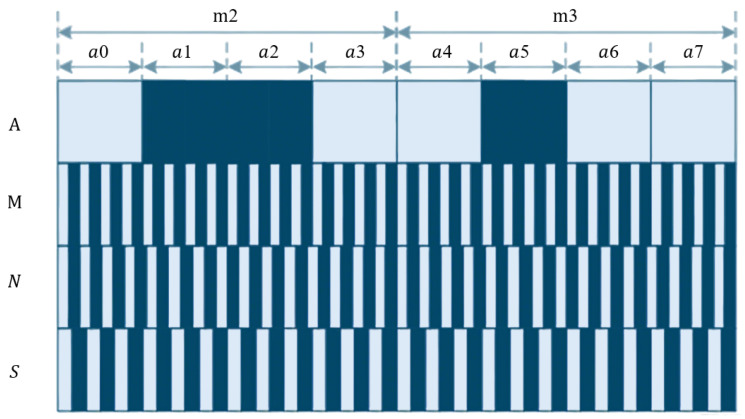
A coding method that combines vernier codes with pseudorandom codes. (Dark blue represents the transparent parts, while light blue represents the opaque parts).

The JFT series of absolute linear encoders, developed by Changchun Yuheng Optics Co., Ltd. (Changchun, China), utilize a coding method that combines vernier codes with pseudorandom codes, as shown in Figure 39. Within each period of the vernier code, a unique absolute position can be identified, which serves as the coarse code. When combined with the fine code obtained through vernier code decoding, real-time position information can be accurately determined. Based on the autocorrelation characteristics of pseudorandom codes, a position cross-verification function has been integrated into the decoding algorithm to ensure accuracy and reliability. The main specifications are as follows: a maximum measuring range of 4040 mm, accuracy of ±3 µm/m or ±5 µm/m, positioning accuracy of ±0.1 µm, a resolution of 0.0025 µm, motion speeds of up to 180 m/min, and communication interfaces including Biss-Closed Loop(BiSS-C) and Data Query(DQ) [52,53].

#### 4.1.3. Encoding Method Combining Absolute and Incremental Tracks

**Figure 40:**
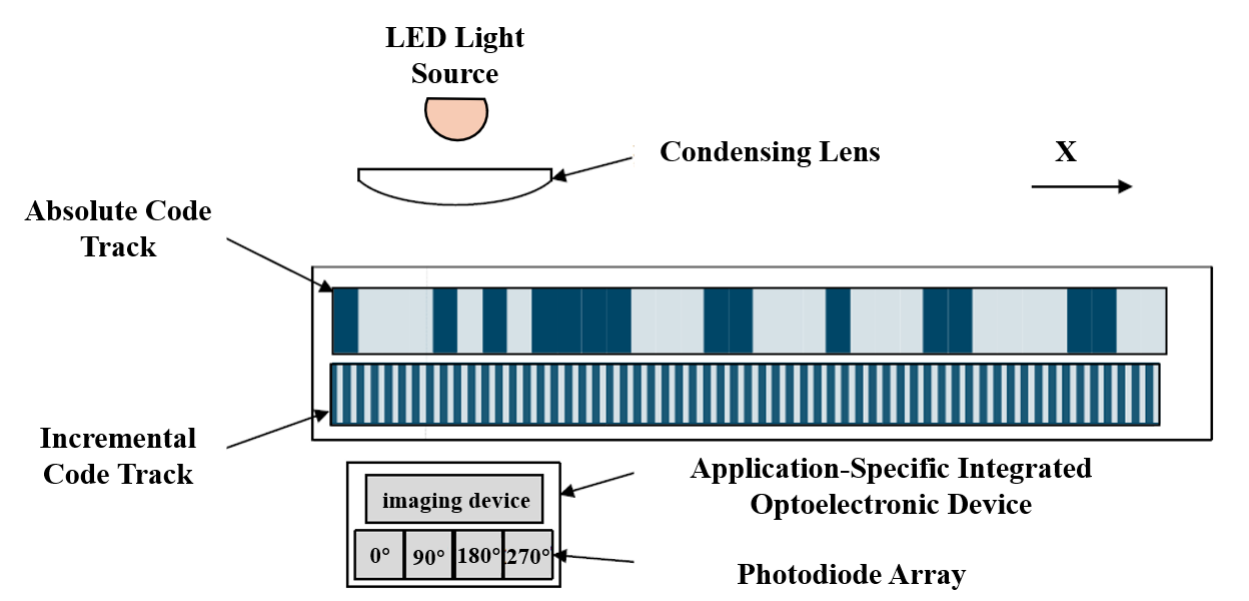
Absolute and incremental codes combined with absolute position coding technology. (Light blue represents the transparent parts, while dark blue represents the opaque parts).

With the ongoing advancement of absolute linear encoder measurement technology, an encoding method that combines absolute and incremental tracks has been adopted in the design of linear encoders. Figure 40 illustrates the measurement principles of an absolute linear encoder that integrates both absolute and incremental tracks. This structure consists of two independent tracks: one incremental and one absolute. The absolute code channel is responsible for the coarse positioning of the absolute position, while the incremental code channel handles fine positioning. The absolute code channel consists of fixed-length markings arranged along the measurement direction. The marked regions are light-transmissive, while the unmarked regions are opaque. The lengths of the markings are non-periodic, and specific combinations of these markings encode the absolute position information. In contrast, the incremental code channel is composed of periodic markings and is primarily used to generate incremental signals [226].

Typically, the photoelectric receiving device for the absolute code channel is a linear-array CMOS or CCD image sensor, which contains several hundred to over a thousand pixel units. Signals are output through a high-speed serial interface, and the signal acquisition time is typically on the order of hundreds of microseconds.

After the absolute encoding region is illuminated by the light source system, the pixels corresponding to the transparent areas appear bright with a higher output voltage, while those corresponding to the opaque areas appear dark with a lower output voltage. As a result, a pattern of alternating light and dark stripes, corresponding to the distribution of irregular markings, is formed on the image sensor. Absolute position information can then be obtained through subsequent circuit-based recognition, processing, and decoding operations.

The photoelectric receiving device for the incremental code channel consists of a photovoltaic cell array, with the number of cells being an integer multiple of 4. Each photovoltaic cell corresponds to an indication grating with a different phase. This section employs the Moiré fringe principle to perform counting and interpolation. The time required for signal acquisition and position calculation is typically around 2 µs.

The absolute position can be obtained by integrating the measurement results from the two components. Upon initial power-up, the system acquires absolute position information by reading the position code. During subsequent operation, the position is calculated by subdividing and counting signals from the incremental code channel, while the absolute code channel is used only for periodic calibration and does not participate in each position acquisition cycle. This significantly reduces the time required to obtain position information.

In summary, the absolute position encoding measurement technology, which combines absolute and incremental code channels, incorporates image processing and coding techniques based on Moiré fringe metrology to enable absolute position measurement. Thanks to these advantages, absolute linear encoders utilizing this technology have been widely adopted in numerical control systems. Representative products include the LC series from HEIDENHAIN, the GA series absolute linear encoders from FAGOR in Spain, and the JC09 model from the Changchun Institute of Optics, Fine Mechanics, and Physics [231].

In 2003, the MITUTOYO Corporation of Japan introduced an absolute linear encoder featuring a three-track multi-channel design, consisting of one incremental track and two absolute tracks. Absolute position measurement is achieved through the encoded combination of the two absolute tracks. This linear encoder offers a maximum resolution of 0.05 µm, accuracy of up to ±3 µm, and a maximum measuring length of 3 m [232].

In 2004, FAGOR Automation of Spain launched a dual-track absolute linear encoder with one incremental track, featuring a grating pitch of 4 µm, and one absolute track. Absolute position measurement is achieved through the combined operation of the two tracks. The product includes two series, with measuring lengths starting from 540 mm and 1020 mm, respectively, and a resolution of 1 µm.

In 2005, HEIDENHAIN of Germany released the LC183 series of absolute enclosed linear encoders, which utilize a glass scale manufactured using the DIADUR graduation process. The encoder features two tracks: an absolute track designed with a single-track pseudorandom code and an incremental track with a grating pitch of 20 µm. The absolute code channel enables absolute position identification through the uniqueness of pseudorandom codes. When combined with the incremental code channel, it allows for high-precision measurements, achieving a resolution of 5 nm and an accuracy grade of ±3 µm, with a maximum measuring length of 1440 mm [228].

**Figure 41:**
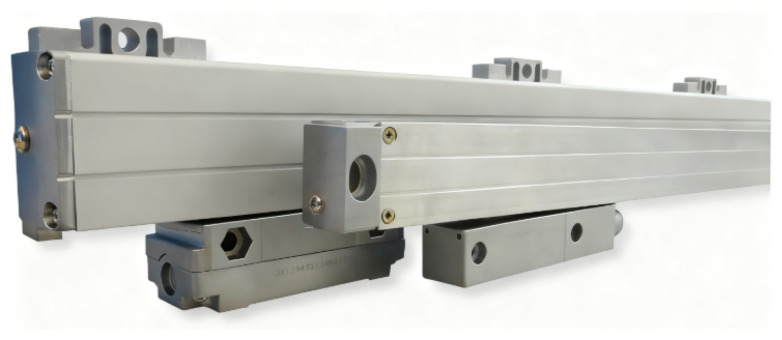
JC09 model absolute linear encoder.

Since 2009, the Changchun Institute of Optics, Fine Mechanics, and Physics, Chinese Academy of Sciences, has operated a dedicated project team for the development of absolute linear encoders. Four years later, the team developed the JC09 one-dimensional absolute linear encoder, as shown in Figure 41. A multi-channel encoding design was adopted by adding an absolute code channel alongside the incremental code channel. Breakthroughs were made in zero-position code design and extraction, as well as in absolute position calculation. Extensive research was carried out on reliability, measurement error compensation, and interpolation. The final product achieves a maximum measurement accuracy grade of ±3 µm, a resolution of 0.05 µm, a maximum measuring range of 3040 mm, and a motion speed of 180 m/min. This product has broken China’s complete reliance on imported absolute linear encoders and has significantly advanced the development of the domestic absolute encoder industry [233,234].

**Figure 42:**
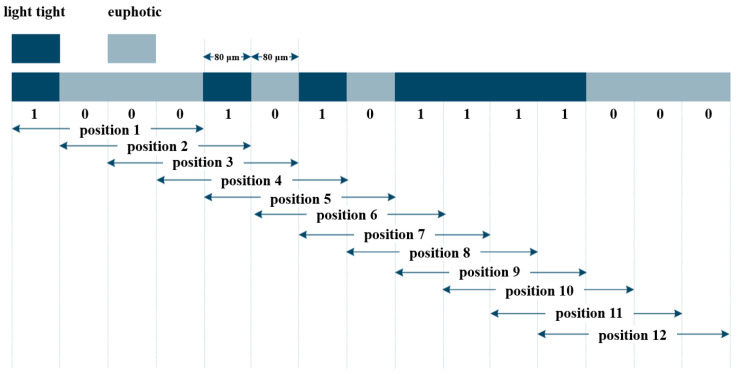
Manchester encoding method.

Building on the research of the JC09 absolute linear encoder, in 2015, Qiao Dong from the Changchun Institute of Optics, Fine Mechanics, and Physics, Chinese Academy of Sciences, used a linear feedback shift register to generate an m-sequence for absolute position encoding and explored the application of Manchester encoding in absolute position encoding. Figure 42 illustrates the position encoding on the absolute linear encoder. As a result, the product achieved enhanced performance, with measurement accuracy of ±8 µm, improved to ±1.5 µm after error compensation, as well as a resolution of 0.01 µm and a maximum operating speed of 180 m/min, thereby bridging the technological gap in the application of domestic linear encoders in closed-loop CNC systems [228].

**Figure 43:**
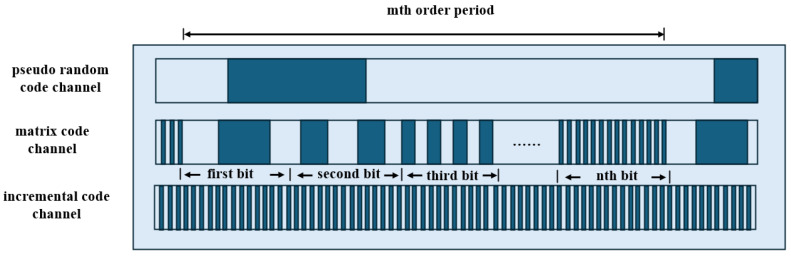
Absolute encoding based on DBCS control theory. (Dark blue represents the transparent parts, while light blue represents the opaque parts).

In 2016, Ding Hongchang from Changchun University of Science and Technology proposed an innovative absolute encoding technique based on DBCS control theory, as shown in Figure 43, integrating matrix codes with pseudorandom codes. A binary tree backtracking algorithm model was established, and the DBCS optimization mechanism was applied—using the patterning process complexity, patterning efficiency, and reading efficiency as weighting functions. Combined with a code channel correction model, this approach enabled the optimal selection of encoding sequences, significantly reducing the complexity of the grating patterning process. Additionally, errors were corrected using the backpropagation (BP) algorithm of artificial neural networks, ultimately achieving a high-precision and highly reliable displacement sensor with a grating displacement measurement error of less than 0.1 µm [229].

### 4.2. Single-Track Absolute Encoding

Against the global backdrop of the continuous iterative development of modern industrial manufacturing and precision measurement technologies, the demand for linear encoders in the direction of integration and digitalization is constantly on the rise. In consideration of the lightweight upgrading of precision instruments and the installation flexibility under complex working conditions, the requirement for the miniaturization of linear encoders is particularly prominent. The encoding method has become a key constraint on the miniaturization of linear encoders. In response, single-track encoding methods have gradually emerged. In the previously discussed multi-track encoding approach, which combines absolute and incremental tracks, the absolute track functions as an independent channel for coarse absolute positioning. Unique position identification is achieved through non-periodic, distinctive encoding, and the encoded information is acquired and decoded using image sensors.

The single-track encoding method enables absolute position measurement through a single track. Its core design is similarly based on the principle of unique position identification using non-periodic, distinctive encoding. The key lies in optimizing the optical system and signal processing algorithms to facilitate accurate encoding readout [87,88]. This encoding method requires only a single track to be inscribed, significantly reducing the sensor size. As a result, with the advancement of absolute encoding technology, single-track encoding has become a key focus of research in the field of absolute linear encoders.

**Figure 44:**
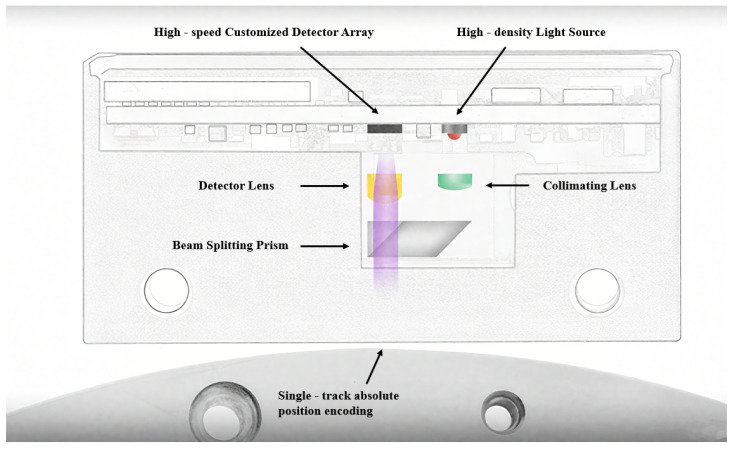
RESOLUTE™ series absolute steel tape encoder.

Renishaw is a leading company in the design of single-track encoders. In recent years, it has introduced the RESOLUTE™ series of absolute linear encoders, as shown in Figure 44, which break away from the traditional framework of Moiré metrology by integrating technologies from multiple disciplines, including microelectronics, mathematical encoding, and high-speed digital signal processing. This enables high-resolution absolute position measurement. The linear encoder features a single-track design that combines absolute position data and built-in phase information into a single code. High-resolution absolute position measurement is achieved solely through absolute position encoding with a minimum line width of 30 µm, supported by an advanced optical system. Encoded image information is acquired at high speed using a 500 Mb/s high-sampling-rate ADC and a custom-designed detector, with the signal then transmitted to a DSP for processing. By integrating high-frequency components and efficient algorithms, the computation time for absolute position information is reduced to just 12 µs. In terms of performance, the system achieves a resolution of 1 nm at a maximum speed of 100 m/s and a 27-bit resolution at a rotational speed of 36,000 rpm, making it suitable for a wide range of applications, including both linear and angular encoding [50,228].

To meet measurement requirements across various scenarios, researchers have developed multiple single-track encoding methods, each featuring distinct encoding rules, signal processing techniques, and application ranges. The following section provides a detailed introduction to several typical single-track encoding methods, analyzing their design principles and technical characteristics.

#### 4.2.1. Displacement Continuous Encoding

**Figure 45:**
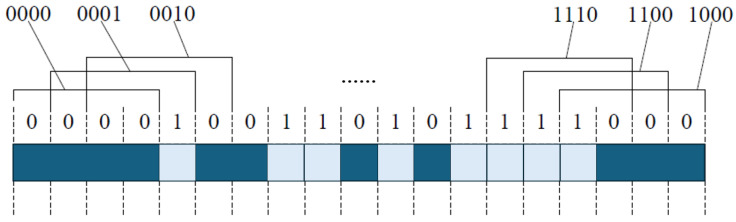
Displacement continuous encoding.

As shown in Figure 45, displacement continuous encoding is a fixed-length binary encoding scheme. The core idea of this algorithm is as follows: for an n-bit code, the all-zero code is used as the initial value. The current code is left-shifted by one bit, with the leftmost bit discarded. A “0” is first appended to the right end, and the resulting code is checked for duplication against existing codes. If no duplication is found, it is accepted as the next code and the left-shift operation continues. If a duplicate is detected, the appended bit is changed to “1” and the check is repeated. If the code is still duplicated, backtracking is performed to adjust the predecessor code: if the last bit of the predecessor is “0”, it is changed to “1”; if the last bit is “1”, backtracking continues to adjust its immediate predecessor. The aforementioned left-shift and backtracking process is repeated until $2^{n}$ unique codes are generated. For an n-bit code, each adjacent pair of codes shares n−1 identical bits. The maximum coding capacity is $2^{n}$, and code uniqueness is guaranteed. For example, a four-bit displacement continuous code sequence may be 0000 1001 1010 1111 000…, where each four-bit code is distinct.

This coding method enables the generation of unique codes using only single-channel marking, which is beneficial for the miniaturized design of sensors. However, there is no explicit rule governing the relationship between code values and position values, making it impossible to directly derive positional information from the code values. A complex decoding process is therefore required. Currently, single-value decoding primarily relies on a lookup table approach, in which the source codes and their corresponding position values are pre-stored in a database, and decoding is performed through query-based matching. However, the table lookup method is associated with issues such as prolonged decoding times and high resource consumption [161].

#### 4.2.2. Pseudorandom Sequence Encoding

Currently, Gray codes, continuous displacement codes, and uniformly distributed absolute codes are all insufficient to meet the demands of single-channel, infinitely long absolute encoding. To address this technical limitation, pseudorandom encoding technology has gradually emerged as a core solution in the field of absolute encoding [54,55,56,57]. Encoding schemes for absolute channels primarily rely on pseudorandom encoding, which employs deterministic algorithms to generate sequences with pseudorandom characteristics. These sequences enable the unique identification of a wide range of absolute positions within a limited code length. Common types of pseudorandom codes include m-sequences, Gold sequences, and R-S sequences. Among them, the m-sequence, as a typical example of linear pseudorandom codes, offers distinct technical advantages. Once the order of the linear feedback shift register is defined, the m-sequence can be used to generate the longest possible binary sequence for that structure, with a period of $2^{n}-1$ (where *n* is the number of register stages), thereby providing greater coding capacity for a given code length.

Moreover, the m-sequence features excellent autocorrelation and shift properties, allowing for rapid decoding through specific synchronization detection and phase comparison algorithms without the need for a predefined codebook index. This capability effectively reduces the storage resource requirements and significantly improves the real-time responsiveness and data processing efficiency of displacement measurement systems [235].

Currently, the typical encoding methods for pseudorandom codes include the following [229].

(1) Single-channel pseudorandom code encoding method: The encoding logic is primarily based on m-sequences, with pseudorandom codes generated using a full-period single-value position function to achieve unique position identification.
(2) Multi-track binary pseudorandom code encoding method: This method extends the single-channel pseudorandom code encoding approach by inscribing multiple pseudorandomly encoded real code tracks onto the linear encoder body. Clear periodic progression exists among the tracks, with the period of each adjacent track decreasing sequentially (i.e., each preceding track has a period that is one unit shorter than the next). Although this method requires a more complex readhead and increases the manufacturing complexity, it allows for the direct output of absolute position values, thereby improving the practicality of the measurement system.
(3) Multi-track p-ary pseudorandom code encoding method: This method is derived from the single-channel pseudorandom encoding approach, with its main enhancement being the expansion of the information capacity by introducing multi-bit encoding to form a p-ary structure. By increasing information redundancy, this multi-valued encoding design effectively reduces the bit error rate during signal transmission and enhances the system’s resistance to interference. Similarly to multi-track binary encoding, it requires a complex readhead, which adds to the manufacturing complexity, but it enables the stable output of absolute position values.
(4) Triple pseudorandom sequence combination encoding method: This method uses a combination of three pseudorandom sequences with cyclic periods $m_{1}$, $m_{2}$, and $m_{3}$ for encoding. The bit lengths of the three sequences are 10 bits, 7 bits, and 3 bits, respectively, corresponding to periods of $2^{10}-1=1023$, $2^{7}-1=127$, and $2^{3}-1=7$. The bit lengths match their respective period values. The specific sequences are as follows:
  - Sequence $m_{1}$: 11111111110000000111000011……11011100111000111000;
  - Sequence $m_{2}$: 111111100001110111100101100……00101000110111000;
  - Sequence $m_{3}$: 1110010.
  By combining multiple sequences, an exponentially expanding encoded sequence can be generated, significantly increasing the coverage range of absolute encoding and enabling absolute position identification in large-scale environments.

Extensive research on pseudorandom sequence encoding has been conducted in domestic academic circles.

**Figure 46:**
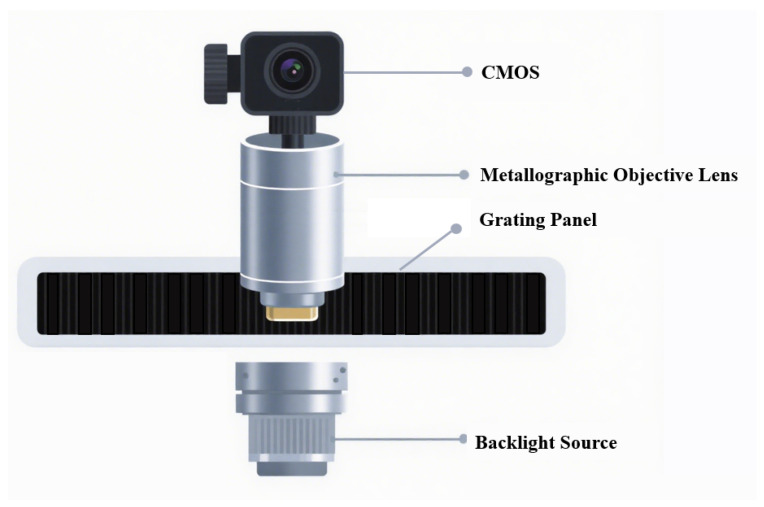
Absolute optical encoder based on a single-track pseudorandom sequence code.

In 2015, the Absolute Linear Encoder Research Team at the Key Laboratory of Micro-Nano Fabrication Technology and Equipment, Guangdong University of Technology, proposed using image recognition technology to directly capture absolute linear encoder codes based on single-track pseudorandom sequence codes (p-element m-sequence pseudorandom binary sequence codes) [236]. As shown in Figure 46, the transient values of the encoding are normally distributed, with a power spectrum that is uniformly spread over a wide frequency band and exhibits favorable correlation characteristics. A unit white bar represents “0”, while a unit black bar represents “1”. Multi-level pseudorandom codes can also be used to enhance the measurement capabilities of the linear encoder. The maximum sequence period is calculated using a specific formula:

(28) $$L_{max}=\prod_{k=0}^{k=1}\left(p^{b}-k+1\right)$$

In the experiment, the absolute linear encoder employing this encoding method achieved a resolution of 769 nm and positioning accuracy of approximately ±1.5 µm.

**Figure 47:**
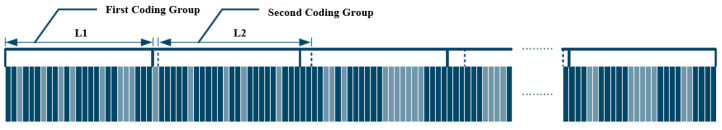
Schematic diagram illustrating the coding principle of two sets of pseudorandom code combinations. (Light blue represents the transparent parts, while dark blue represents the opaque parts).

In June 2024, Geng Yong from Xi’an University of Technology proposed an absolute linear encoder measurement system based on dual CCDs, as shown in Figure 47. This system employs an encoding method that combines two sets of pseudorandom codes, allowing more absolute positions to be represented with fewer code elements. The generation of m-sequences is also required. By applying a fusion encoding technique based on two sets of m-sequences, the system improves the absolute position resolution. Additionally, the multi-phase fast Fourier transform (MPFFT) algorithm is used to perform phase subdivision on a single Moiré fringe. Within a measurement range of 4 cm, the system achieves an absolute position resolution of 9.92 µm, with an incremental displacement measurement error of less than 0.11 µm [237].

Most existing research has focused on optimizing the application of m-sequence pseudorandom codes, such as improving the encoding stability by enhancing sequence generation circuits and reducing environmental noise interference through optimized decoding algorithms. However, there is a lack of fundamental theoretical research in areas such as the design of novel pseudorandom codes and multi-sequence fusion encoding. The m-sequence has inherent limitations; its maximal sequence encoding is uniquely determined by a given initial value, meaning that only one maximal sequence can be generated from each initial condition. This uniqueness restricts the flexibility to select the optimal encoding scheme based on practical conditions such as the grating engraving precision and signal reading environment. To some extent, this limitation in encoding selection hinders further improvements in the measurement accuracy and operational reliability of linear encoders.

#### 4.2.3. Hybrid Encoding

To overcome the limitations of single encoding methods and fully exploit the strengths of various encoding forms, hybrid encoding has been developed. By flexibly selecting the aforementioned encoding types, it creates a composite encoding scheme that incorporates multiple technical advantages.

The absolute code–simplified code method, as a hybrid encoding technique, effectively combines the coarse error resistance of Gray code with the single-channel benefits of m-sequences. Technically, Gray code features only a one-bit binary difference between adjacent code groups, which significantly reduces the likelihood of coarse errors during signal reading. Meanwhile, m-sequences, as pseudorandom sequences, offer a single-channel encoding structure that facilitates simplified sensor design. The absolute code–simplified code method capitalizes on these features to achieve the complementary integration of their respective advantages.

The core structure of this encoding method consists of several sets of base codes, each comprising two functionally distinct components: a rolling code and a reference code. The rolling code performs a dynamic identification function by generating a unique binary sequence that changes with displacement during the rolling cycle, thereby enabling precise position differentiation. The reference code, in contrast, serves as a baseline unit, maintaining a fixed repetitive sequence throughout the rolling process. Its primary role is to provide synchronization calibration or an initial positioning reference for signal reading. Rather than being excluded from the design, it plays a fundamental role as a reference element.

However, this encoding method has specific limitations in the signal extraction process: it must be used in conjunction with a slit structure, where the encoded information is retrieved through the modulation of the optical signal by the slits. Moreover, the number of bits in the absolute code directly determines the number of slits and the corresponding configuration of photoelectric conversion elements. For each additional bit, an extra set of slits and photoelectric elements is required. This one-to-one correspondence leads to a significant increase in the structural complexity and physical size of the sensor as the bit count rises. As a result, this encoding method is not well suited for high-bit-count grating displacement sensors and presents clear limitations in applications demanding high precision and a wide measurement range [226].

**Figure 48:**
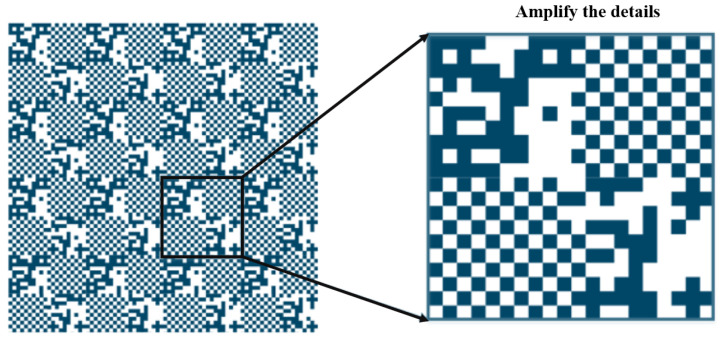
Partial encoding pattern of the two-dimensional absolute position. (Dark blue represents the light-transmitting parts, and white represents the light-opaque parts).

Except for the absolute simplified code, as shown in Figure 48, a two-dimensional absolute position measurement technique based on hybrid encoding was proposed by Niu Ruru and colleagues from Hefei University of Technology. By combining pseudorandom sequences (m-sequences) with binary sequences, an absolute code channel was constructed and further integrated with a checkerboard incremental code channel to form a dual-channel hybrid encoding pattern. Encoded images were captured using a CMOS camera, followed by deflection correction through template matching. A “coarse” position was then obtained via single-channel decoding. Subsequently, “fine” position compensation was achieved by extracting edges using a bilateral model sub-pixel subdivision algorithm. This method ultimately achieved a resolution of 0.1 µm and average measurement accuracy of ±0.8 µm within a measurement range of 80 mm × 80 mm [238].

## 5. Discussion of Errors in Absolute Linear Encoder Measurement Technology

**Figure 49:**
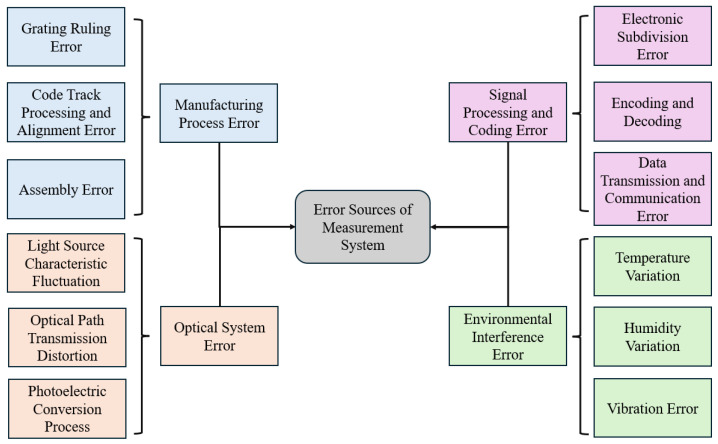
Sources of error in the linear encoder measurement system.

As a core component of high-precision displacement measurement, the absolute linear encoder plays a critical role in determining the technological capabilities of fields such as high-end manufacturing and precision metrology. With the accuracy requirements for the linear encoder workpiece stage reaching the sub-nanometer level, more stringent demands have been placed on error compensation within displacement measurement systems. Consequently, it is essential to analyze and compensate for the various sources of error present in the linear encoder displacement measurement system [29,239,240,241,242,243,244,245]. Manufacturing errors can arise from microstructure fabrication processes and alignment tolerances [60,206,246]. Optical system errors relate to interferometer alignment, lens aberration, and wavefront distortion [18,43]. The sources of error in the linear encoder measurement system are illustrated in Figure 49.

In this paper, the main error sources are ranked based on their compensability and impact, as presented in Table 2. This ranking enables readers to quickly grasp the relative importance of different error sources.

### 5.1. Errors Related to Manufacturing Processes

As a core component for high-precision displacement measurement, absolute linear encoders rely on the nano-scale scribing accuracy and manufacturing consistency of complex grid structures [247]. Manufacturing process errors mainly stem from non-ideal factors in the processes of grating scribing, code track machining, and assembly and can be specifically classified into the following categories.

#### 5.1.1. Grating Line Inscription Errors

As the reference element of the measurement system, the quality of the grating line inscription sets the theoretical upper limit of measurement accuracy. Grating ruling errors refer to the deviation of the actual ruling position from the ideal one. Based on their manifestations, grating ruling errors can generally be classified into three categories [248]:

(1) Curvature errors, characterized by the rulings appearing in a curved distribution;
(2) Yaw angle errors, where the actual rulings are not parallel to the ideal ones but intersect within the same plane;
(3) Positional errors, indicating a displacement between the actual and ideal ruling positions.

These errors directly impact key performance metrics of the grating, including the resolving power, ghost line suppression, stray light level, and diffraction quality.

In the photolithographic projection manufacturing process, distortion of the projection objective is a key factor contributing to ruling errors. Such distortion causes the nonlinear deformation of the rulings, leading to both curvature and positional errors. Whether the distortion is positive or negative, the severity of a curvature error increases significantly with the degree of distortion.

To address this issue, the distortion parameters of the objective lens can be optimized and installation errors strictly controlled to effectively reduce grating line errors and enhance the grating manufacturing accuracy. Additionally, grating line errors are influenced by a combination of factors, including the precision of the manufacturing equipment and the level of processing technology. For high-density grating lines on the linear encoder, the maximum manufacturing error is typically maintained within ±2 µm. If the error exceeds this range, the image acquisition quality of the linear array grating will be directly compromised, thereby affecting the displacement measurement accuracy of the linear array grating system [249]. During the actual manufacturing process, line breakage may also occur. Broken grating lines can obstruct light transmission and negatively impact the measurement accuracy. However, thanks to the operational characteristics of the photoelectric receiver in the linear encoder—capable of simultaneously receiving light projected from dozens of grating lines and leveraging the “averaging effect” of Moiré fringes—the precision fluctuations caused by the breakage of a single grating line can be significantly reduced [250].

#### 5.1.2. Channel Processing and Alignment Errors

The operation of multi-channel absolute linear encoders relies on the coordinated performance of multiple channels. In particular, in configurations that combine absolute and incremental channels, the absolute channel performs coarse positioning, providing a general positional framework for measurement, while the incremental channel is responsible for fine positioning, enabling high-resolution position determination within the coarse range defined by the absolute channel. Therefore, precise alignment between the two channels is essential to ensure that the positional information that they provide is accurately synchronized.

When the deviation in parallelism between code tracks becomes excessive, a mismatch can occur between the absolute position and the subdivided position, which severely compromises the measurement accuracy. As the measurement length increases, this error tends to accumulate, posing a significant challenge for long-range and high-precision measurement tasks. Therefore, during the manufacturing and installation of multi-track absolute linear encoders, it is crucial to maintain strict control over the parallelism of the code tracks. This requires the use of high-precision machining techniques and meticulous installation and calibration procedures to minimize the negative impacts of track misalignment on the measurement results.

Although single-track absolute linear encoders eliminate the complex alignment issues inherent in multi-track systems, the machining accuracy of the internal encoding pattern within the track remains equally critical. Encoding patterns such as pseudorandom codes and displacement continuous codes require extremely high engraving precision. These patterns convey positional information through unique permutations and combinations, so any machining deviation in a single code element can alter the overall meaning of the code and lead to decoding errors. By adopting advanced processing technologies (such as holographic exposure technology, the wet chemical development process, metallic glass nanowire positioning technology, nano-imprint lithography technology, and electron evaporation) and strict quality control measures, the processing errors of single-code-track coding patterns can be effectively reduced, and the measurement performance of the linear encoder can be improved [251,252,253,254,255].

#### 5.1.3. Assembly Errors

**Figure 50:**
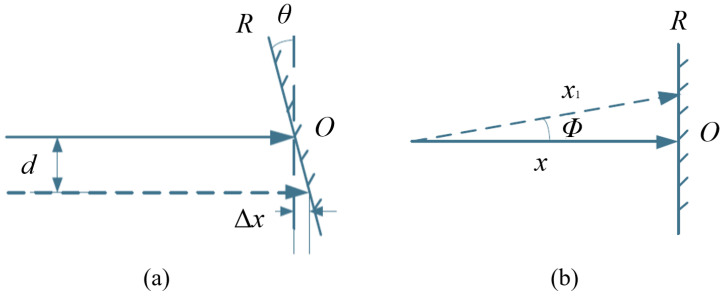
Assembly error. (**a**) Abbe error. (**b**) Cosine error.

In practical applications of absolute linear encoders, the installation accuracy has a direct impact on the precision of the measurement results [256,257,258]. As a major source of full-range positional error, installation errors primarily consist of two types, Abbe errors and cosine errors [259,260], as illustrated in Figure 50. Their occurrence is closely related to the spatial arrangement of the linear encoder within the machine tool and the relative positioning of the moving components [261].

An Abbe error is a typical installation error resulting from the misalignment between the measurement axis and the motion axis of the workpiece. According to the Abbe principle, accurate measurement requires that the axis of the measuring instrument be collinear with the motion axis of the workpiece. Otherwise, any deflection of the moving component will introduce additional measurement errors. In a CNC machine tool, if the measurement axis of the linear encoder is parallel but not coincident with the motion axis of the worktable, and the worktable has a deflection angle θ, the Abbe error can be calculated using the following formula:

(29) Δx=d·tanθ

where *d* is the distance between the two axes, and θ is the deflection angle of the worktable. In practical applications, when d=100 mm and θ=0.01°, the Abbe error can reach 17.45 µm, which is significant in high-precision machining scenarios. The extent of the Abbe error is positively correlated with both the distance between the two axes and the deflection angle. To mitigate this error, two measures should be taken: (1) during installation, the linear encoder should be positioned as closely as possible to the machining point to minimize the value of *d*; (2) the structural rigidity of the machine tool and the straightness of the guideways should be enhanced to reduce the deflection angle θ of the worktable, thereby fundamentally minimizing the cumulative error caused by deflection.

A cosine error results from the misalignment between the measurement axis of the linear encoder and the motion axis of the worktable. When there is an angle ϕ between the two axes, a deviation arises between the measured value and the actual displacement. The calculation formula is as follows:

(30) $$\Delta x=x_{1}-x=x\left(\frac{1}{\cos\phi }-1\right)$$

where *x* is the actual displacement of the worktable, and ϕ is the angle between the two axes. For example, when x=1000 mm and ϕ=0.1°, the cosine error is approximately 1.52 µm. Although this is smaller than the Abbe error under the same conditions, it still needs to be controlled in high-precision measurements. The magnitude of the cosine error depends on both the displacement and the angle, and its control relies on precision adjustments during installation. By ensuring the straightness of the scale grating and the scale housing, as well as maintaining the parallelism between the installation surface of the linear encoder and the machine tool guideway, the angle ϕ can be minimized, thereby reducing the impact of the error [169,228]. During the calibration and measurement process of the linear encoder, the high-bandwidth nano-positioning platform can assist in achieving more precise position localization, thereby improving the overall measurement accuracy of the absolute linear encoder [262].

### 5.2. Optical System Errors

As the core component responsible for converting code channel information into optical signals, the optical system of an absolute linear encoder is subject to errors, primarily arising from three critical sources [239]: fluctuations in light source characteristics, distortions in the optical transmission path, and deviations in the photoelectric conversion process.

In terms of light source characteristics, intensity fluctuations, wavelength drift [263], and insufficient beam uniformity are the primary sources of error. These factors directly compromise the stability and consistency of the optical signal, introducing fundamental interference into subsequent signal processing.

Errors during optical path transmission are primarily caused by defects in optical components and misalignment of the optical path, both of which degrade the imaging quality [12]. Lens distortion can result in the stretching or compression of the code channel image, leading to symbol position recognition errors in image processing-based decoding systems. Astigmatism causes the light spot to focus at different positions along different directions, resulting in the asymmetric distribution of the Moiré fringe signal and significantly increasing the magnitude of errors in the subdivision process.

In addition, the relative positional deviations of optical components introduced during the assembly phase of the measurement system—such as misalignments in the installation of lenses, laser sources, gratings, readheads, and photoelectric receivers—can cause the actual propagation path of the measurement beam to deviate from the ideal trajectory, thereby further degrading the imaging quality and measurement accuracy. Chang et al. introduced the concept of “fused-like angles” as a replacement for conventional roll–pitch–yaw representations, effectively improving the stability and interpretability of six-DOF grating interferometers [77].

Errors in the photoelectric conversion stage primarily arise from the characteristics of the devices and environmental disturbances, including the non-uniform response of photodiode arrays, dark current drift, and signal crosstalk. Variations in the response of photodiode arrays can lead to imbalances in the amplitudes of quadrature signals; when the imbalance exceeds a certain threshold, subdivision errors tend to increase significantly. As a temperature-sensitive parameter, dark current introduces a DC offset that severely undermines the stability of the signal’s zero point, exerting a systematic impact on the measurement results.

For the elimination of errors in optical systems, errors can first be suppressed through structural design. Specifically, light source fluctuations are isolated via a dual-beam symmetric optical path, and optical path distortion is isolated through beam parallelism constraints—both measures serve to reduce the introduction of errors. Subsequently, residual errors are compensated for using algorithms. It is essential to quantify the direction and magnitude of the impacts of various errors on the measurement results and to clearly differentiate between “offsettable errors” (e.g., drift that can be eliminated by differencing) and “calibration-required errors” (e.g., constant offset) [264,265,266].

### 5.3. Signal Processing and Encoding Errors

The signal processing and encoding stage of absolute linear encoders is responsible for converting optical signals into position information. The associated errors primarily arise from electronic interpolation, encoding and decoding, and data transmission, as detailed below.

#### 5.3.1. Electronic Interpolation Errors

Electronic interpolation is a core technique for achieving high resolutions in grating measurement systems [267]. It works by digitally processing and interpolating the sinusoidal and cosinusoidal electrical signals generated from the Moiré fringes of the grating, allowing the original fringe period to be divided into a greater number of equal segments. This effectively overcomes the limitations of mechanical engraving precision. However, the accuracy of the interpolation is directly affected by three critical factors: signal quality, noise interference, and the performance of the interpolation algorithm [268]. In practical applications, these factors can lead to non-negligible position measurement errors. When higher-order harmonics are present in the Moiré fringe signal, the nonlinearity of the sinusoidal waveform can cause subdivision errors, leading to significant positional deviations. The RESOLUTE absolute encoder from Renishaw, combined with a precision optical system, minimizes electronic subdivision errors to ultra-low levels, thereby delivering high performance with a resolution of up to 1 nm, a measuring range of up to 5 m, and accuracy of 1 µm/m [237].

#### 5.3.2. Encoding and Decoding Errors

Encoding and decoding errors are two fundamental types of errors in absolute optical grating-based position measurement systems. These errors arise, respectively, from the processes of encoding design and physical implementation and from signal acquisition and information interpretation, directly impacting the measurement accuracy and system reliability.

Encoding errors primarily result from the design of encoding rules and the physical fabrication of code tracks. First, inherent limitations in encoding rules can introduce errors. For example, in natural binary encoding, multiple bits may change simultaneously between adjacent intervals, increasing the likelihood of bit conflicts. In matrix encoding, the division of code tracks into sectorial regions makes the system highly sensitive to assembly precision; axial vibrations or installation misalignments can directly cause positional deviations in the code tracks. Second, defects in physical implementation can also lead to errors [269]. These include inconsistent carryover caused by insufficient engraving precision (e.g., line engraving errors in natural binary encoding leading to incorrect codes), as well as issues such as deviations in track parallelism in multi-track designs and code pattern distortion due to the complexity of fabricating pseudorandom sequences in single-track designs.

Decoding errors arise from the processes of signal acquisition and information processing. On one hand, errors may be introduced due to sensor recognition asynchrony, such as the response delay of photoelectric receivers to multi-bit varying signals in multi-channel encoding, or the degradation of the signal-to-noise ratio caused by uneven illumination or excessively large detector areas during the coordinated multi-channel measurement of vernier codes. On the other hand, errors can result from decoding algorithms and procedures, including conversion errors when transforming Gray code into natural binary code, time and resource consumption issues in displacement continuous encoding using lookup tables, accuracy limitations in synchronization detection and phase comparison algorithms during pseudorandom sequence decoding, and subdivision errors in binary barcode decoding caused by Fourier transforms, phase calculation, and the diffusion of quantization errors.

In addition, in the hybrid encoding of absolute and incremental code channels, variations in the time required by the image sensor to acquire absolute code channel information may lead to dynamic decoding errors due to insufficient real-time performance.

#### 5.3.3. Data Transmission and Communication Errors

The position information of absolute optical encoders is transmitted to subsequent devices via serial communication protocols (such as EnDat, SSI, BiSS, etc.). Errors during data transmission and communication mainly arise from deficiencies in protocol mechanisms, electrical interference, and system compatibility issues. At the protocol level, the synchronization accuracy between the clock signal and the data signal directly affects the measurement reliability. For example, in the EnDat protocol, timing deviations between the falling edge of the clock signal and data reading can result in position sampling errors. The SSI protocol, lacking the ability to dynamically configure parameters, is susceptible to errors caused by mismatches between preset parameters and the actual operating conditions. Electrical noise is another critical source of error. In high-speed transmission scenarios, signal attenuation and electromagnetic interference (EMI) can still lead to data frame loss or distortion. Additionally, the protocol conversion process (such as the HEIDENHAIN protocol conversion module for EnDat and FANUC protocols) may introduce additional latency and conversion errors.

In addition, the signal delays of different channels in a multi-track absolute linear encoder vary, which can lead to asynchronous measurement. This, in turn, results in measurement errors and synchronous measurement errors [270]. In the high-speed motion of absolute linear encoders, it is necessary to track the dynamic phase shift corresponding to displacement changes in real time. Traditional static phase calculation methods are prone to measurement errors due to “phase lag” [271]. The Hybrid Input–Output (HIO) algorithm can achieve the fast tracking of the phase with displacement by introducing “negative feedback adjustment” and optimizing the iteration step size, thereby reducing phase delay errors during high-speed motion [272].

### 5.4. Environmental Interference Errors

Environmental factors exert complex and random effects on absolute optical encoders, primarily including the temperature, humidity, and vibration, as detailed below [273].

Temperature variation is the most significant source of environmental error, with its impact reflected in three main aspects. The first is the thermal expansion of the scale—based on the linear expansion coefficient of the metal scale, temperature fluctuations can cause considerable positional errors [274]. The second is the relative thermal expansion and contraction between the optical encoder and the connected machine tool—positioning errors may arise due to the different linear expansion coefficients of materials such as cast iron and steel plates. Third, the parameters of electronic components drift with temperature. For instance, the offset voltage drift of an operational amplifier can introduce measurement errors.

Temperature errors are generally linear and can be reduced through compensation. For precision machining, it is essential to maintain a stable workshop temperature to minimize the impact on the machining accuracy. Additionally, the absolute linear encoder should be positioned away from heat sources to reduce thermal effects on the encoder. The temperature during the bonding process of the scale grating must also be controlled, typically maintained at 20 °C. If the temperature is not stable, the length of the scale grating for encoders with the same measuring range may vary during factory calibration, which can create difficulties for CNC machine tool manufacturers in implementing effective temperature compensation [275,276].

Humidity fluctuations can cause hygroscopic expansion in non-metallic scales (such as glass scales). Contaminants such as oil or dust adhering to the scale surface can obstruct light transmission, resulting in a reduction in signal amplitude. In severe cases, this may lead to the failure of absolute code channel decoding. The linear encoder can be housed within a sealed environment filled with nitrogen gas, which not only prevents dust from entering but also extends the service life of the circuitry [277].

During the feed and cutting processes of a machine tool, vibrations are generated. When transmitted to the scale body and slider of the linear encoder, these vibrations can cause both components to vibrate as well, leading to fluctuations in the scale’s readings and resulting in vibration-induced errors. If the linear encoder is exposed to sustained and intense vibrational loads, it may fail to operate properly. Therefore, the linear encoder should be mounted on the most rigid part of the machine tool and must not be installed on hollow or low-rigidity components. To avoid resonance, the structural rigidity of the linear encoder should also be enhanced—for instance, by increasing the number of fastening screws between the scale and the CNC machine, integrating the scale housing with its support structure, or increasing the wall thickness of the housing. These measures help to raise the natural frequency of the linear encoder, ensuring that it remains well above the operating frequency of the machine tool.

In response to issues such as cavity alignment deviation, interference signal drift, and noise sensitivity, studies have proposed the use of cavity-based femtosecond laser sensing methods to improve alignment tolerance and suppress errors, including the measurement of Fabry–Perot cavity displacement based on mode-locked lasers [278], the measurement of the cavity length using the improved peak-to-peak method [279], and the measurement of the absolute angles of broadband solid-state cavities [280]. These methods demonstrate the potential to enhance error suppression and stability in high-precision encoders.

## 6. Summary and Outlook

### 6.1. Research Summary

As a core component for high-precision displacement measurement in advanced manufacturing, the development of absolute linear encoders is closely tied to the autonomous capabilities of key sectors such as intelligent manufacturing, precision metrology, and semiconductor processing. This study presents a systematic investigation into the measurement technologies of absolute linear encoders, outlining their technological evolution, their fundamental principles, and the current state of development both domestically and internationally. The main research findings are summarized as follows.

In terms of the technical framework, this paper summarizes the foundational theoretical system for absolute linear encoder measurement, covering three primary measurement principles based on Moiré fringes, diffraction gratings, and imaging techniques. Among these, the Moiré fringe method has become the conventional mainstream approach due to its magnification effect and error averaging characteristics; the diffraction grating method enables nanometer-level resolutions through the interpolation of interference signals; and the imaging-based method surpasses the resolution limits of traditional techniques by utilizing digital image processing algorithms to achieve sub-pixel positioning.

In addition, this paper introduces the encoding and decoding technologies of absolute linear encoders: it elaborates on traditional absolute encoding methods such as natural binary codes and Gray codes and serial communication protocols including EnDat and SSI, as well as electronic subdivision and image-based decoding methods. It clearly identifies the key performance indicators of absolute measurement, which include the resolution, accuracy, measurement range, and response speed, among others.

In encoding research, quasi-absolute linear encoder encoding achieves a balance between precision and cost through the design of an “incremental code channel + aperiodic auxiliary code channel”. In this context, non-embedded encoding employs a parallel structure consisting of an independent reference code channel and an incremental code channel, while embedded encoding integrates reference information into the incremental code channel to achieve structural unification.

Absolute linear encoder encoding can be classified into multi-channel and single-channel types. Multi-channel encoding is based on the core design principle of “multi-channel coordination”, enabling absolute position measurement through the parallel arrangement of multiple functionally distinct code channels. Based on the encoding format, it can be further categorized into natural binary encoding, Gray code, matrix encoding, vernier code, and hybrid formats that combine absolute code channels with incremental code channels. Single-track encoding is based on the core design principle of “single-track integration”, in which all absolute position information is carried by a single track. Depending on the encoding method, it can be classified into hybrid encoding, displacement continuous encoding, and pseudorandom sequence encoding.

Furthermore, in this paper, the errors within the linear encoder measurement system are categorized into four distinct types: manufacturing process-related errors, optical system errors, signal processing and encoding errors, and environmental interference errors. A comprehensive analysis of the causes underlying each type of error is presented, thereby providing a theoretical foundation for precision improvement and the formulation of compensation strategies.

### 6.2. Future Prospects

As high-end manufacturing continues to demand greater measurement accuracy, wider measuring ranges, improved dynamic performance, and enhanced environmental adaptability [281], absolute linear encoder technology is advancing toward higher precision, greater integration, and increased intelligence. In response to the current technical bottlenecks and development needs, future research may focus on the following areas.

The encoding method is a key determinant of the performance of absolute linear encoders. Currently, multi-track encoding is hindered by structural complexity and alignment difficulties, while single-track encoding encounters challenges related to decoding efficiency and resistance to interference over long measuring ranges. Future research should prioritize the development of novel encoding schemes that offer high uniqueness, strong fault tolerance, and ease of fabrication. On the one hand, pseudorandom encoding techniques based on advanced theories such as chaotic sequences and quantum encoding could be explored to improve the capacity and contamination resistance of single-track systems. On the other hand, deep learning algorithms could be integrated to optimize the track recognition logic [282,283], enabling the robust decoding of blurred images through neural networks. This would reduce the reliance on optical imaging quality and support stable measurements in harsh industrial environments.

The demand for miniaturized high-precision linear encoders is becoming increasingly high in fields such as semiconductor manufacturing and micro–nano operations. The current separation between optical systems and signal processing circuits hinders further integration. Future breakthroughs may be achieved through the following approaches.

Wafer-level optical fabrication processes can be employed to integrate the scale grating, imaging lens, and photodetector onto a single chip, thereby reducing optical path losses and assembly errors.

MOEMS-based miniature readheads can be developed, using micro-mirror arrays and waveguide structures to reduce the size of the optical system.

The on-chip integration of signal processing circuits can be advanced by incorporating high-speed ADCs, DSPs, and communication interfaces into dedicated chips, thereby improving the data processing efficiency and system stability.

## Figures and Tables

**Table 1:** Comparison of performance indicators for closed absolute optical encoders.

Performance Metric	HEIDENHAIN	MITUTOYO	FAGOR	CIOMP
Precision Class	±3 μm	±3 μm	±3 μm	±3 μm
Movement Velocity	Up to 600 m/min	Up to 180 m/min	Up to 180 m/min	Up to 120 m/min
Resolution	1 nm	0.005 μm	0.1 μm	0.01 μm
Maximum Measurement Length	4240 mm	3000 mm	3040 mm	1200 mm
Data Interface	EnDat 2.2	Fanuc, Mitsubishi	SSI, FreeDat	CPE-Bus

**Table 2:** Table of importance ranking for error sources.

Error Source	Compensability	Degree of Impact on Measurement	Ranking
Manufacturing process error	Partial errors can be compensated for through the averaging effect and algorithms; however, reticle defects and mechanical geometric errors are fundamental errors that cannot be completely eliminated.	Determine the upper limit of the system’s theoretical accuracy and serve as irreversible hardware-level error sources.	1
Environmental interference error	Errors can be reduced through temperature control, real-time compensation, and vibration isolation measures, but complete control is difficult to achieve.	Affect randomness and long-term stability in actual industrial environments, impact measurement repeatability and drift, and act as the main obstacle to application scenario adaptability.	2
Optical system error	Errors can be significantly reduced with high-quality optical components and assembly calibration.	Directly affect fringe contrast and signal symmetry, leading to orthogonal signal distortion and increased subdivision errors, and serve as a key bottleneck for system-level accuracy stability.	3
Signal processing and encoding error	Limited by hardware resolution and sampling rate.	Mainly affect high-resolution and high-speed measurement scenarios and represent error sources with the highest controllability, relying on algorithm optimization.	4

## Data Availability

The data presented in this study are available on request from the corresponding author.
